# Antagonistic interactions safeguard mitotic propagation of genetic and epigenetic information in zebrafish

**DOI:** 10.1038/s42003-023-05692-3

**Published:** 2024-01-05

**Authors:** Divine-Fondzenyuy Lawir, Cristian Soza-Ried, Norimasa Iwanami, Iliana Siamishi, Göran O. Bylund, Connor O´Meara, Katarzyna Sikora, Benoît Kanzler, Erik Johansson, Michael Schorpp, Pierre Cauchy, Thomas Boehm

**Affiliations:** 1 Department of Developmental Immunology, Max Planck Institute of Immunobiology and Epigenetics, Freiburg, Germany; 2 Department of Medical Biochemistry and Biophysics, Umeå University, Umeå, Sweden; 3 Bioinformatic Unit, Max Planck Institute of Immunobiology and Epigenetics, Freiburg, Germany; 4 Transgenic Mouse Core Facility, Max Planck Institute of Immunobiology and Epigenetics, Freiburg, Germany; 5 Institute for Immunodeficiency, Center for Chronic Immunodeficiency (CCI), University Medical Center, Faculty of Medicine, University of Freiburg, Freiburg, Germany

**Keywords:** Developmental biology, Genetics, Immunology

## Abstract

The stability of cellular phenotypes in developing organisms depends on error-free transmission of epigenetic and genetic information during mitosis. Methylation of cytosine residues in genomic DNA is a key epigenetic mark that modulates gene expression and prevents genome instability. Here, we report on a genetic test of the relationship between DNA replication and methylation in the context of the developing vertebrate organism instead of cell lines. Our analysis is based on the identification of hypomorphic alleles of *dnmt1*, encoding the DNA maintenance methylase Dnmt1, and *pole1*, encoding the catalytic subunit of leading-strand DNA polymerase epsilon holoenzyme (Pole). Homozygous *dnmt1* mutants exhibit genome-wide DNA hypomethylation, whereas the *pole1* mutation is associated with increased DNA methylation levels. In *dnmt1*/*pole1* double-mutant zebrafish larvae, DNA methylation levels are restored to near normal values, associated with partial rescue of mutant-associated transcriptional changes and phenotypes. Hence, a balancing antagonism between DNA replication and maintenance methylation buffers against replicative errors contributing to the robustness of vertebrate development.

## Abstract (web summary)

Genetic interaction studies in zebrafish larvae using hypomorphic alleles of dnmt1 and pole1 indicate that impaired DNA replication is associated with increased levels of post-replicative DNA methylation.

## Introduction

DNA methylation is a key repressive epigenetic mark that modulates gene expression and prevents genome instability^1–6^. After replication of the DNA template during S phase, cell type-specific DNA methylation patterns on the parental strands are copied onto the newly synthesized daughter strands^7,8^ by the maintenance methylase Dnmt1 (refs. ^9,10^), although a contribution of the de novo methylases Dnmt3a and Dnmt3b particularly for repetitive elements has also been reported^11^. In general, methylated cytosine occurs most frequently in CG dinucleotides; however, in certain cell types, such as mouse germinal vesicular oocytes, non-CG sites (summarily referred to as CH) are also heavily methylated^12^.

Parental DNA methylations patterns at palindromic CG sites are restored in a biphasic pattern; the first phase appears to be coupled to the presence of Dnmt1 at the replication fork, whereas the second phase is not coupled to replication and continues throughout the cell cycle^3,13–25^. Whereas Dnmt1 has strong preference for hemi-methylated CG sites that are generated during replication, it has little if any activity on non-CG sites^12^. Rather, partially (CHG) or non-palindromic (CHH) sites (H representing A, C, or T) are targeted by the de novo methylation activity of Dnmt3a and Dnmt3b (refs. ^26–28^), which have intrinsically different site preferences^29^. Dnmt1 indirectly contributes to the establishment and maintenance of methylation at non-CG sites, since most non-CG methylation is associated with heavily methylated arrays of CG sites^28,30–33^.

Interestingly, the treatment of cells with DNA synthesis inhibitors is associated with DNA hypermethylation^34^, possibly mediated by de novo enzyme activity. This observation reinforces the notion of an inverse relationship between replication and maintenance DNA methylation. To overcome the potential limitations of studies using cell lines, we set out to analyze this epistatic relationship at the levels of global DNA methylation patterns, transcriptome composition, and phenotypes in developing zebrafish larvae.

## Results

### Design of the study

**Fig. 1:**
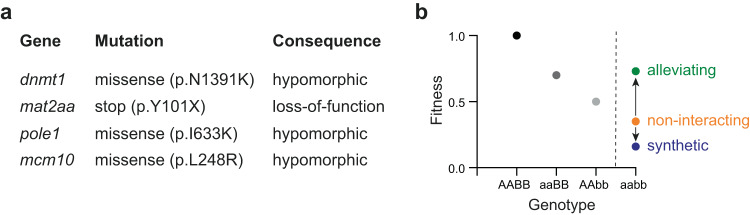
Study design. **a** List of mutant alleles examined in this study. **b** Schematic representation of genetic interaction analysis. The fitness (as expressed in particular phenotypic characteristics) of genotypes determines whether genetic interaction is alleviating (when the phenotype is less severe than expected from the phenotypes of single mutants) or synthetic (when the phenotype is more severe than expected). When the phenotype conforms to expectation, then a non-interacting situation is recorded (see Methods for details of the multiplicative model used for the present analysis).

The present study rests on the identification of viable mutants in genes that play pivotal roles during DNA replication and DNA methylation (Fig. 1a). Our previous studies have extensively characterized a hypomorphic allele of zebrafish *dnmt1* (refs. ^35,36^). In contrast to embryonic lethality of *dnmt1* null alleles^37,38^, the hypomorphic allele of *dnmt1* (p.N1391K) identified in our screens has surprisingly little effect on overall survival, development and fertility, with the exception of a severely impaired lymphoid development^35,39,40^; nonetheless, the lethal phenotype of *dnmt1* null alleles^37,38^ identifies *dnmt1* as a central node in establishing and maintaining proper DNA methylation patterns and suggests that other DNA methylase enzymes possess limited compensatory capacity. For comparative purposes, we included a null allele of *mat2aa* (p.Y101X)^40^. *mat2aa* and its paralog *mat2ab* encode rate-limiting non-redundant enzymes in the biosynthesis of the methyl group donor S-adenosyl-methionine (SAM)^41^; hence, in contrast to the *dnmt1* mutant, the *mat2aa* mutant is expected to exhibit non-specific effects on all cellular processes requiring SAM as cofactor, including, but not limited to, DNA methylation. Fish homozygous for a null allele of *mat2aa* exhibit a severe phenotype, resulting in death 6–7 days after fertilization (dpf)^40^.

Two additional mutants were used in the present study to examine whether interference with DNA replication affects DNA methylation levels. To this end, we identified and characterized hypomorphic alleles of *pole1* (p.I633K) and of *mcm10* (p.L248R). Pole1 encodes the catalytic subunit of DNA polymerase epsilon that is considered to be the main DNA polymerase involved in leading strand synthesis; it is a member of the B family of DNA polymerases^42^ and highly related to DNA polymerase delta, which functions in lagging strand synthesis^43^. Fish homozygous for the hypomorphic *pole1* (p.I633K) allele survive into the third week after fertilization^39,40^ (see below). *mcm10* encodes minichromosome maintenance protein 10, which is required during both initiation and elongation phases of DNA replication^44^. Homozygosity of the mutant *mcm10* allele examined here is associated with stunted growth, but mutant fish can survive for several weeks^40^ (see below).

Our analyses were conducted at 5 dpf, when all four mutants are viable. For each mutant (Fig. 1a), we characterized the extent of DNA methylation, the composition of the larval transcriptome, and the extent of T cell development (at times complemented by the analysis of retinal features). Moreover, we generated all 6 pairwise combinations of mutants and used the three parameters to assess the presence and types of genetic interaction (Fig. 1b). In general, epistasis is considered when the phenotype of a double-mutant organism deviates from the expected neutral phenotype. Positive (alleviating) genetic interaction often occurs when two genes function in the same pathway such that the phenotype is less severe than expected; negative (synthetic) interaction often indicates the activity of genes in parallel pathways affecting the same aspect of the phenotype which is more severe than the neutral expectation (Fig. 1b).

### Characterization of a hypomorphic *pole1* allele

**Fig. 2:**
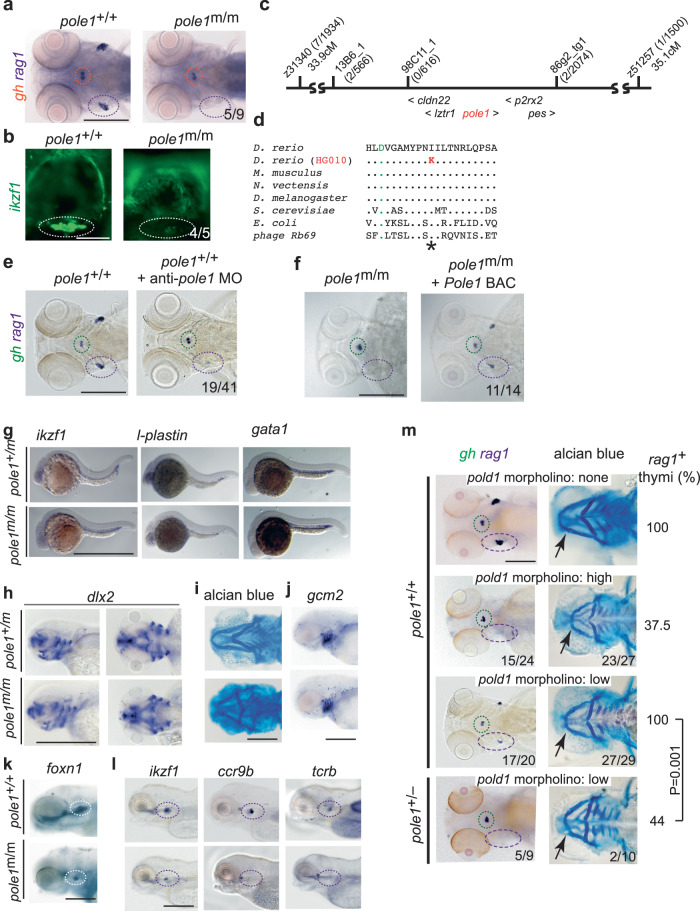
Characterization of a zebrafish *pole1* hypomorphic allele. **a** Diagnostic whole-mount RNA in situ hybridization using *rag1* (thymus; purple circle), and *gh* (hypophysis; orange circle) of zebrafish embryos at 5 days after fertilization (dpf) (dorsal view; representative of 9 wild-type/mutant pairs; all wild-type fish exhibit strong *rag1* signals, whereas 5 mutant fish lack a *rag1* signal; 4 mutant fish have weak *rag1* signal; see also Fig. 9). **b** Thymic lymphopenia revealed by a *ikzf1:EGFP* reporter (lateral views; representative of 5 wild-type/mutant pairs; all wild-type fish exhibit strong signals, whereas 4 mutant fish have a minimal signal (as shown); 1 mutant fish lacks clearly discernible signal). **c** Genetic map for the region on chromosome 5 in the vicinity of the *pole1* gene (not to scale; transcriptional orientations indicated). The number of recombination events observed between several genetic markers and the mutated locus in the number of meioses shown is indicated in brackets. **d** Identification of an I633K missense mutation in the *pole1* gene of HG010 mutants. Partial protein sequences encompassing the region of the first of three catalytic aspartates (asterisk; orange font) and the universally conserved isoleucine (I) residue in family B DNA polymerases (red outline) are shown for several species (Genbank accession numbers): *D. rerio* Pole1, amino acids [aa] 623-643 (NP_001121995); *M. musculus* Pole1, aa 624-644 (AAD46482); *N. vectensis* DNA Pole, aa 610-620 (XP_001628405); *D. melanogaster* DNA Pole, aa 622-642 (BAB17608); *S. cerevisiae* POL2, aa 638-658 (CAA63235); *E. coli* polB, aa 417-437 (YP_851261); *bacteriophage Rb69* DNA polymerase ChainA, aa 409-429 (1WAJ_A). **e** Phenocopy of impaired T cell development in *pole* morphants; in 19 embryos, the *rag1* signal was specifically lost (the *gh* signal was unchanged), otherwise only slightly reduced or normal. **f** Phenotypic rescue by mouse *Pole1*; in 11 of 14 mutants, *rag1* signals could be detected after BAC injection. **g** Normal embryonic haematopoiesis in *pole1* mutants, as determined by whole-mount RNA in situ hybridization at 24 h after fertilization with the indicated gene-specific probes; representative of between 4 and 7 wild-type/mutant pairs. **h** Near normal craniofacial structures as determined by whole-mount RNA in situ hybridization with *dlx2*, at 3 days after fertilization (dpf) (representative of 5 wild-type/mutant pairs). **i** Analysis of craniofacial structures as determined by alcian blue staining to visualize cartilage structures at 4 dpf (representative of 3 wild-type/mutant pairs). **j** Normal shape of pharyngeal ectoderm indicated by g*cm2* expression; representative of 5 wild-type/mutant pairs. **k** Normal pharyngeal endoderm indicated by *foxn1* expression; representative of 5 wild-type/mutant pairs. **l** Impaired T cell differentiation indicated by expression of *ikzf1, ccr9b* and *tcrb* (representative of between 5 and 15 wild-type/mutant pairs). **m** Genetic interaction between *pole* and *pold1* (representative embryos shown). First row: Uninjected wild-type (*pole*^+/+^) embryos are shown for reference. The *gh* signal is marked in green, the *rag1* signal in the thymus in purple (whole mount RNA in situ hybridization, left panel); the arrow points to the intersection of cartilaginous structures of the ethmoid plate of the neurocranium and the mandibular arch (alcian blue staining; right panel). The fraction of embryos with *rag1*-positive thymi is shown in the right-hand column. Second row: Injection of high concentrations of anti-*pold1* oligonucleotides (1 mM) results in the loss of *rag1* signals in the majority of embryos (15/24). The mandibular arch is shorter in 23 out of 27 morphants. Third row: Injection of low concentrations of anti-*pold1* oligonucleotides (0.2 mM) results in milder phenotypes; 17 out of 20 morphants had a reduced *rag1* signal in the thymus; the mandibular arch was normal in 2 and somewhat smaller in 27 out of 29 morphants. Fourth row: Injection of low concentrations of anti-*pold1* oligonucleotides (0.2 mM) into embryos heterozygous for the *pole*^t20320^ allele caused severe phenotypes. In 5 out of 9 morphants, no *rag1* signal was observed, whereas it was present but reduced in four fish; the mandibular arch was severely affected in 2 (representative embryo shown), mildly affected in 6 and normal in 2 morphants. The effect of anti-*pold1* oligonucleotides on *rag1* expression in wild-type versus *pole*^*+/*t20320^ heterozygotes is statistically significant (t- test; two-tailed). Scale bars, (**a**, **e**–**m** [1 mm]; **b** [0.25 mm]).

The HG010 mutant line (allele designation *pole1*^t20320^) was identified in the Tübingen 2000 forward genetic screen among a collection of approximately forty mutants lacking or exhibiting severely reduced numbers of *rag1*-expressing cells in the thymus^39,40,45^ (Fig. 2a); heterozygous carriers are indistinguishable from wild-type siblings. As determined by RNA in situ hybridization, the thymic rudiment of homozygous mutants still contains some *rag1*-positive haematopoietic cells, but normal numbers of growth hormone (*gh*)-expressing cells in the hypophysis, indicating the tissue-specificity of the mutant phenotype. The lymphopenic mutant thymus is also revealed using an *ikzf1:EGFP* transgene^46^ that marks all lymphocytes (Fig. 2b). The mutation exhibits an autosomal recessive mode of inheritance and maps to chromosome 5 (Fig. 2c); for the five genes mapping to this interval (*cldn22*, *p2rx2*, *pole1*, *lztr1*, *pes1*), wild-type sequences were recorded for the exon sequences and the associated splice junctions for all genes, except for *pole1*. A missense mutation was found in exon 17 of the *pole1* gene at residue 2043 (T > A; Genbank accession number NM_001128523) causing the replacement of an evolutionarily conserved isoleucine residue by lysine (p.I633K); the mutated amino acid residue occurs C-terminal of the first of three aspartates forming the catalytic triad of the polymerase domain (Fig. 2d). To support the assignment of *pole1* as the candidate gene of the HG010 mutant line, two additional tests were carried out. Injection of an anti-sense morpholino directed against sequences encompassing the initiation codon of the *pole1* mRNA phenocopied the defects of homozygous mutants (Fig. 2e). The injection of a BAC genomic clone containing the wild-type mouse *Pole1* gene rescued *rag1* expression in zebrafish *pole1* mutants (Fig. 2f), indicating an evolutionarily conserved function of the mouse gene in supporting thymopoiesis in the transgenic embryos. Interestingly, lack of *pole1* activity does not perturb embryonic haematopoiesis per se, as indicated by the normal expression patterns of *ikzf1*, *l-plastin* and *gata1* in mutant embryos at 24 hpf (Fig. 2g). However, pharyngeal cartilage structures are abnormal, as assessed by *dlx2* expression (Fig. 2h) and alcian blue staining (Fig. 2i), reflecting the detrimental impact of the mutant Pole1 on rapidly proliferating cells types; by contrast, the formation of pharyngeal ectoderm and endoderm appears to proceed normally, as assessed by the expression patterns of *gcm2* (Fig. 2j) and *foxn1* (Fig. 2k), respectively. As expected, the lymphoid precursor compartment in the thymus is severely affected; larvae lack expression of two markers of T cell precursors (*ccr9b* and *ikzf1*), and of a marker of immature T cells (*tcrb*) (Fig. 2l).

DNA polymerase delta is considered to be the major polymerase for lagging strand synthesis^43^, and is believed to functionally replace DNA polymerase epsilon^47^. To further assess the functional importance of the Pole1(p.I633K) mutation, we examined the genetic interaction of the *pole1*^t20320^ allele with *pold1*, using *pold1* gene-specific anti-sense oligonucleotides^48^. To this end, we first examined the phenotype of *pold1* morphants; as expected, injection of high concentrations of the morpholino caused the loss of *rag1*-expressing cells (Fig. 2m). By contrast, injection of low concentrations reduced, but did not eliminate T cell development in the morphants; however, when the same low concentration of anti-*pold1* morpholino was injected into embryos heterozygous for the *pole1*^t20320^ allele, developing thymocytes were no longer detectable in the majority of embryos and craniofacial defects became readily apparent (Fig. 2m). This result is indicative of a synthetic genetic interaction (see Fig. 1b), suggesting that the two genes independently affect the same pathway.

To examine whether the p.I633K mutation may represent a temperature-sensitive allele, we compared the phenotypes of homozygous mutants raised at 28.5 °C or 24 °C (Supplementary Fig. 1a–d). The results indicate that in contrast to *gh-*expressing cells in the hypophysis, T cells developing in mutants are much more sensitive to the temperature shift, best appreciated in the plot of the signal ratios (Supplementary Fig. 1d). In order to substantiate the temperature-sensitivity of this mutation, it was engineered into pol2 of *S. cerevisiae* (p.I648K) and into mouse Pole1 (p.I634K), respectively. The yeast variant grew well at 22 °C, but failed to grow at 37 °C (Supplementary Fig. 1e). The presence of the mutated mouse *Pole1* gene led to early lethality, exhibiting a drastic shift in Mendelian ratios already at the blastocyst stage (Supplementary Fig. 1f). To examine whether the POLE1 mutation in patients diagnosed with FILS syndrome^49^ would be viable in mice, we engineered the equivalent splice-site mutation into the mouse *Pole1* locus (Supplementary Fig. 2a). The homozygous mutant genotype is severely disadvantaged from the morula stage onwards; no live homozygous mutant mice were born (Supplementary Fig. 2b). In the yeast model, a suppressor mutation in pol2 (p.D657H) was identified that restored the capacity to grow at 37 °C (Supplementary Fig. 2c, d). However, engineering the equivalent mutation into the mouse *Pole1* gene (p.S643H) did not improve the survival rate of mice homozygous for the p.I634K/S643H double mutation (Supplementary Fig. 2e). Collectively, these results precluded the use of a mouse *Pole1* mutant for epistasis analysis with a viable *Dnmt1* mutant allele^36^.

An insufficient capacity for replicative DNA synthesis in the mutants could explain the abnormalities in rapidly proliferating cells, such as developing thymocytes and those forming the head cartilage. Perturbations in the S phase of the cell cycle may activate a p53-dependent checkpoint that causes cells to enter the apoptotic pathway. To examine this hypothesis, we performed a genetic interaction analysis using a *p53* mutant zebrafish line^50^; however, this analysis is complicated by the fact that zebrafish *pole1* and *p53* genes reside on the same chromosome in close proximity. Nonetheless, the one recombinant obtained in this experiment exhibits a much milder phenotype than the *pole1* single mutant (Supplementary Fig. 3a, b). To confirm this observation, we injected a *p53* gene-specific antisense morpholino^48^ into *pole1* homozygous mutants. Remarkably, significant numbers of thymocytes were found in the morphants (Supplementary Fig. 3c); moreover, an almost complete normalization of the craniofacial phenotype was also achieved (Supplementary Fig. 3d).

### Characterization of a hypomorphic *mcm10* allele

The recessive mutation in the IG335 mutant line (allele designation *mcm10*^t23336^) was localized to chromosome 4 using meiotic recombination mapping (Supplementary Fig. 4a) by virtue of the significantly reduced number of *rag1*-expressing haematopoietic cells in the thymus at 5 dpf (Supplementary Fig. 4b). Sequencing of the six genes in the critical interval (*phyh*, *ucmaa*, *mcm10*, *nudt5a*, *cdc123*, *camk1da*) identified a thymidine to guanine (T to G) transversion in the second nucleotide of codon 248 in exon 5 of the *mcm10* gene (ENSDARG00000045815) (p.L248R) as the only detectable aberration. Three additional experiments confirmed the correct identification of the mutated gene. The injection of a *mcm10*-specific anti-sense morpholino directed against *mcm10* exon 4 splice acceptor site replicated the phenotype (Supplementary Fig. 4c), whereas the *mcm10* mutant phenotype was partially rescued by injection of mouse *Mcm10* mRNA and a BAC clone containing the wild-type mouse *Mcm10* gene, respectively (Supplementary Fig. 4d, e). The mutated residue L248 is evolutionarily conserved (Supplementary Fig. 4f) and is located in the ID domain of Mcm10, which binds to single-stranded DNA and Polα during initiation of DNA replication in a competitive manner^51,52^. L248, together with four other hydrophobic amino acids (I302, L303, V339, L341) forms the β3 and β5 plated sheets in the ID domain of Mcm10; hence, the p.L248R mutation is predicted to alter the interaction with DNA and Polα and hence impair DNA replication. When examined at 24 h post fertilization (hpf), lack of wild-type *mcm10* (Supplementary Fig. 4g) does not affect embryonic haematopoiesis as indicated by normal expression of *cmyb*, *scl*, *gata1*, and *l-plastin*. The formation of pharyngeal ectoderm, as assessed by the expression patterns of *gcm2* appears to be normal; mesodermal cartilage is only mildly affected, as assessed by *dlx2* expression and alcian blue staining (Supplementary Fig. 4h). By contrast, intra-thymic T cell development is strongly reduced in *mcm10* mutants when assessed at 5 dpf, as seen in whole-mount RNA in situ hybridizations with probes specific for *cmyb*, *ikzf1*, and *tcrb* (Supplementary Fig. 4i). Survival tests showed that 10–15% of IG335 mutants with the p.L248R missense mutation survive until 3–4 weeks of age, although they grow more slowly and can thus be recognized by their significantly smaller body size and malformed eyes (Supplementary Fig. 4j). *mcm10* mutants which survive until 5 weeks of age are developmentally retarded, have fewer or no circulating red blood cells and thus appear pale (Supplementary Fig. 4j). The thymus is small and hypocellular as revealed by haematoxylin-eosin staining, with no detectable haematopoietic cells expressing the *rag 1* gene compared to the wild-type thymus; haematoxylin-eosin staining of kidney sections revealed that the kidney (the site of myelopoiesis and B cell lymphopoiesis) contains only few haematopoietic cells and no *rag1*-expressing cells (Supplementary Fig. 4k). The impaired Mcm10 activity appears to activate the p53 pathway, since a profound phenotypic rescue of the *mcm10* mutant phenotype was observed in *mcm10/p53* double mutants (Supplementary Fig. 3e, f).

### Methylation status in single and double mutants

**Fig. 3:**
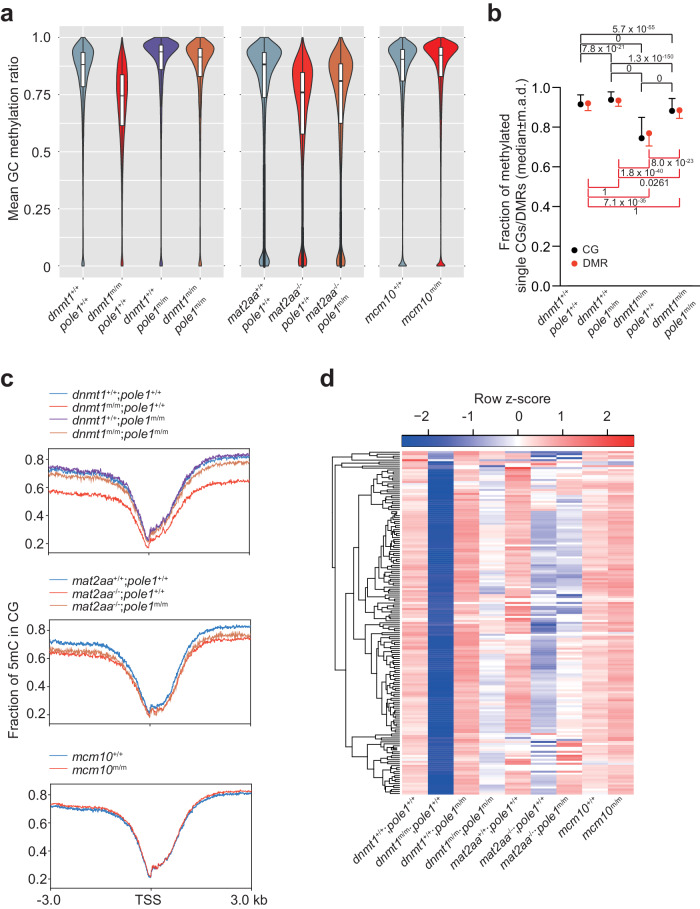
Global changes in DNA methylation patterns in single and double mutant zebrafish. **a** Global methylation patterns of CG dinucleotides expressed as mean methylation ratios; median±m.a.d. **b** Quantification of data in (a) represented as median±m.a.d.; DMR, differentially methylated regions. The *P* values for pair-wise comparisons (t-test; two-sided) are indicated, corrected for multiple testing. **c** Methylation levels around transcriptional start sites (TSS). **d** Variable methylation levels of 222 differentially methylated regions (as assessed by CG methylation) present in *dnmt1* mutants across different genotypes.

**Fig. 4:**
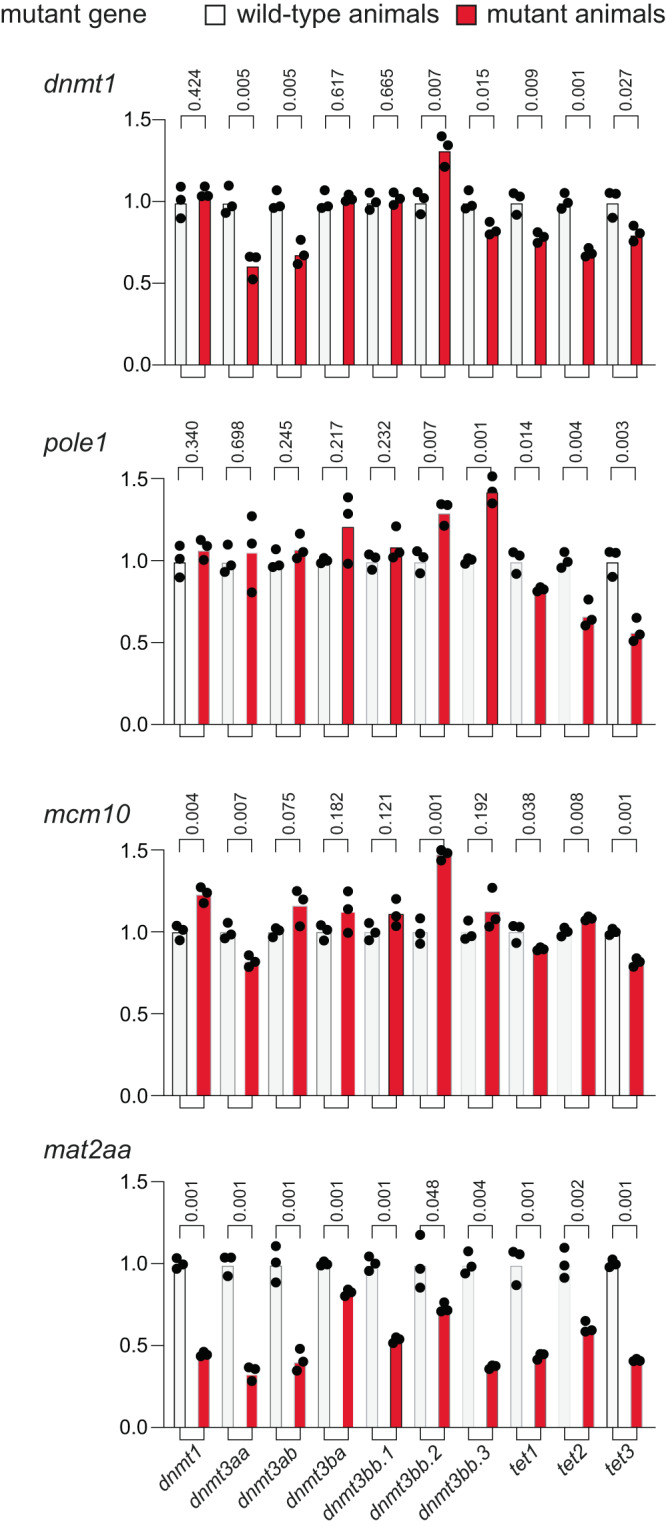
Expression analysis of genes associated with the DNA methylation process. qPCR analysis was performed on 5 dpf embryos of wild-type and mutants (*n* = 3 for each genotype) for the four mutant lines; mean values are indicated. The *P* values (t-test; two-tailed) for pairwise comparisons of each wild-type (white columns)/mutant (red columns) group are indicated above the columns.

To provide a baseline for subsequent epistasis analyses, we first examined the DNA methylation status in the four single mutants by whole genome bisulfite sequencing (WGBS). In *dnmt1* mutants, overall CG methylation levels are substantially decreased, when measured at individual dinucleotides and differentially methylated regions (Fig. 3a, b; Supplementary Fig. 5). This reduction is also seen at transcription start sites (TSS), with differences becoming more pronounced with increasing distance from TSS (Fig. 3c). As expected, CG methylation levels are also reduced in *mat2aa* mutants (Fig. 3a; Supplementary Fig. 6). By contrast, CG methylation levels are increased in *pole1* mutants (Fig. 3a–c; Supplementary Fig. 7), whereas only a modest increase of genome-wide CG methylation levels occurs in *mcm10* mutants (Fig. 3a, c; Supplementary Fig. 8), possibly in line with their milder phenotype compared to *pole1* mutants (Fig. 2; Supplementary Fig. 4). We attribute the increased methylation levels in the DNA replication mutants to the extended time available to the Dnmt1 enzyme to complete post-replicative methylation of nascent strands, and, as judged from transcriptional profiles of genes encoding components of the DNA methylation process (Fig. 4), to increased de novo DNA methylation activity, possibly including such activity of Dnmt1 itself^53,54^.

CG methylation is widely recognized as the major form of DNA methylation; however, genome-wide studies have uncovered the presence of non-CG, i.e., CHG and CHH methylation (H representing A, C, or T) in vertebrate genomes^12^. Intriguingly, stem cells and post-mitotic cells, such as those in the brain, exhibit elevated levels of non-CG methylation;^12^ dnmt3-like enzymes are thought to be largely responsible for generating and maintaining methylation patterns at non-CG sites^26–29^. Our experimental model provided an opportunity to assess the fate of non-CG methylation patterns under conditions of impaired Dnmt1 and/or Pole1 activities. Specifically, we asked (i) whether the extent of non-CG methylation, like that of CG methylation, is reduced in embryos homozygous for the hypomorphic *dnmt1* allele and is increased in *pole1* mutants; (ii) whether methylated sites are lost and/or new sites become methylated in the mutant embryos; and (iii) whether methylation changes are similar between CG and non-CG methylation sites. Using a modified version of the snakePipes WGBS package (see Methods), we determined the patterns of CG, CHG, and CHH methylation, and plotted non-zero methylation levels for sites shared among each group of embryos, that is, comparing the corresponding wild-type patterns against *dnmt1* and *pole1* single and double mutants (group 1); *mat2aa* and *pole1* single and double mutants (group 2); and *mcm10* mutants (group 3).

**Fig. 5:**
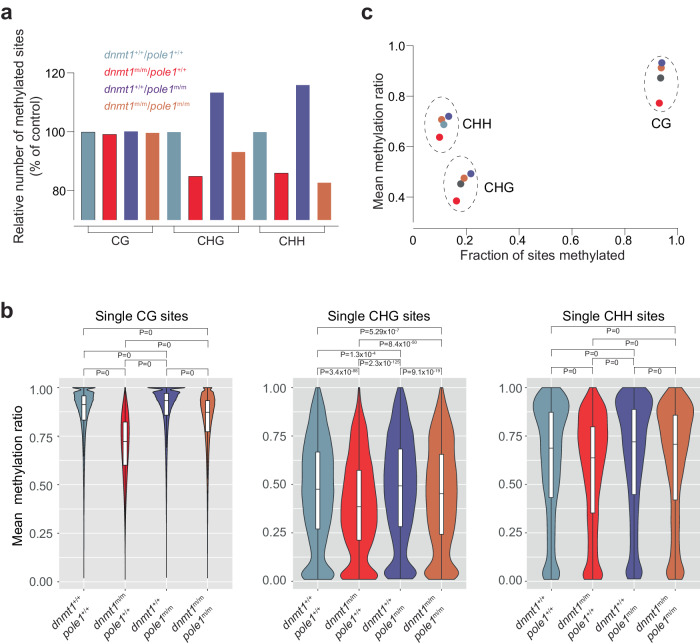
Alterations in non-CG methylation patterns in *dnmt1* and *pole1* mutants. **a** Variable numbers of methylated sites for CG dinucleotides, and CHG and CHH tri-nucleotides. The number of sites in wild-type fish is set to 100 (for absolute numbers of total and methylated sites, see Supplementary Table 1). **b** Violin plots of methylation ratios; median±m.a.d. The significance levels of the differences between mean methylation ratios are indicated (t-test, two-tailed; corrected for multiple testing). **c** Correlation plot between the fractions of sites methylated and their mean methylation ratios. For **b**, **c**, color code for genotypes indicated in **a**.

When restricting the analysis to methylated CG sites in the *dnmt1*/*pole1* intercross, the results are qualitatively similar to the analysis that considered both zero and non-zero CG sites (see Fig. 3), as expected from the high percentage of methylated CG sites in the genome. Although the different genetic constellations only minimally affected the absolute number of methylated CG sites (Fig. 5a), it profoundly affected the extents of methylation at these sites (Fig. 5b), indicating that the dominant changes in methylation patterns for CG sites are quantitative (Supplementary Table 1). Of note, about three-quarters of the *dnmt1* mutant-induced loss in mean methylation ratio is restored in the *dnmt1/pole1* double mutants (Fig. 5b; Supplementary Table 1; Supplementary Fig. 9), possibly explained by the fact that the hypomorphic Dnmt1 enzyme^36^ has more time to target hemi-methylated sites that are generated after semi-conservative DNA replication directed by the hypomorphic Pole1 enzyme.

By contrast, additional qualitative changes are observed for CHG and CHH sites. Many of the partially palindromic CHG sites become demethylated in *dnmt1* mutants, indicating that Dnmt1 is directly or indirectly involved in non-canonical methylation (Fig. 5a, b; Supplementary Table 1); correspondingly, many new CHG sites acquire methylation in the *pole1* mutant condition (Fig. 5a, b; Supplementary Table 1), either through de novo activity of Dnmt1 and/or increased activity of Dnmt3-like enzymes (Fig. 4). Overall, the changes in the extents of methylation at CHG sites follow the pattern seen for CG sites; lower in *dnmt1* mutants, higher in *pole1* mutants, and intermediate in double mutants (Fig. 5a, b; Supplementary Table 1). For non-palindromic CHH sites, we observed that the decrease of mean methylation ratios in *dnmt1* mutants is less pronounced than for CG and CHG sites, and is even maintained above the wild-type levels in the double mutants (Fig. 5a, b; Supplementary Table 1), as a consequence of the *pole1* mutant-induced hypermethylation. These findings suggest that Dnmt1 is less important in the generation and/or maintenance at non-palindromic CHH sites.

In sum, the analysis of CG, CHG, and CHH sites indicates that although they are differentially affected by the loss of wild-type activities of *dnmt1* and *pole1*, reduced activity of Dnmt1 decreases both CG and non-CG methylation levels accompanied by a relative shift to methylation at CHH sites (Fig. 5c). In addition, impaired DNA replication is associated with a higher degree of de novo methylation (Fig. 5c), possibly associated with increased expression of some members of the *dnmt3* family of genes (Fig. 4). A comparable result is observed in *mcm10* mutants, wherein an increased number of methylated sites is accompanied by only small increases in mean methylation ratio (Supplementary Fig. 10; Supplementary Table 1); in this context, it is notable that the increase in *dnmt3*-like gene expression levels is less pronounced in the *mcm10* mutant than in the *pole1* mutant (Fig. 4).

**Fig. 6:**
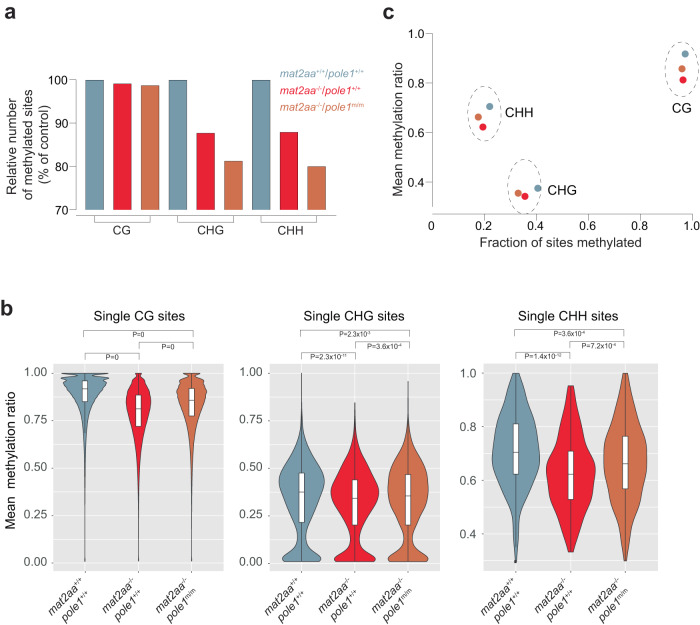
Alterations in non-CG methylation patterns in *mat2aa* and *pole1* mutants. **a** Variable numbers of methylated sites for CG dinucleotides, and CHG and CHH tri-nucleotides. The number of sites in wild-type fish is set to 100 (for absolute numbers of total and methylated sites, see Supplementary Table 1). **b** Violin plots of methylation ratios; median±m.a.d. The significance levels of the differences between mean methylation ratios are indicated (*t*-test, two-tailed; corrected for multiple testing). **c** Correlation plot between the fractions of sites methylated and their mean methylation ratios. For **b**, **c**, color code for genotypes indicated in **a**.

A similar analysis for *mat2aa* mutants resulted in a different outcome. Most notable is the drastic loss of methylated sites, most pronounced for CHH; remarkably, this loss is even accentuated in the double mutants (Fig. 6a; Supplementary Table 1); the diminished mean methylation ratios of the remaining sites in *mat2aa* mutants are partially rescued in the *mat2aa/pole1* double mutants (Fig. 6b, c; Supplementary Table 1; Supplementary Fig. 11), although the extent of restoration is smaller than that in *dnmt1/pole1* double mutants. Overall, the perturbed methylation landscape as a result of the *mat2aa* mutation is most likely due to the pleiotropic effects of reduced intracellular SAM levels, particularly with respect to impaired compensatory activity of de novo methylases (Fig. 4).

### Transcriptional landscapes in single and double mutants

The observation that the combination of reduced catalytic activities of Dnmt1 and Pole1 results in a compensatory interaction that alleviates the effects of the individual mutations on the DNA methylation patterns prompted us to examine other aspects of their phenotypes. To this end, we examined the whole-body transcriptomes at 5 dpf (a time point at which all mutants are viable), initially focusing on *dnmt1* and *pole1* single and double mutants.

**Fig. 7:**
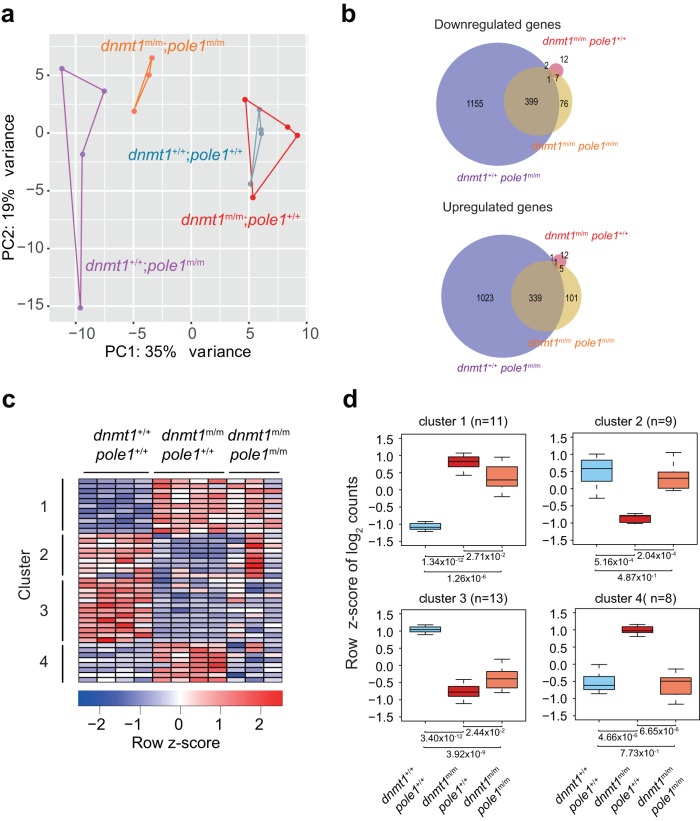
Transcriptional landscapes in *dnmt1* and *pole1* mutants. **a** Dynamic changes in transcriptomes of *dnmt1* and *pole1* single and double mutants, as indicated by principal component analysis. **b** Venn diagram indicating the overlap of differentially expressed genes for down-regulated and up-regulated genes in fish of the indicated genotypes. **c** Four clusters of co-regulated genes are identified in *dnmt1* mutant fish; each column represents a biological replicate. **d** Co-regulation of groups of genes in (c) identifies 2 gene clusters with restored wild-type levels in *dnmt1/pole1* double mutants; box plots (mean; box, upper/lower quartile; whiskers, minimal/maximal values).

The profiles are largely overlapping for wild-type and *dnmt1* single mutants (Fig. 7a); indeed, a mere 41 differentially expressed genes were observed in this two-way comparison (Fig. 6b, c; Supplementary Fig. 12). Of note, although perturbed DNA methylation has been linked to genome instability^1,2^, homozygosity of the *dnmt1* allele used here does not lead to the transcriptional signature associated with p53 activation (Supplementary Fig. 12); this is in line with the finding that inactivation of the *p53* gene does not alleviate the phenotypic changes of the *dnmt1* mutation (Supplementary Fig. 3g). By contrast, the *pole1* mutation is associated with drastic changes of the transcriptome (Fig. 7a, b); nearly 3000 genes are differentially expressed in *pole1* single mutants, including the signature of an upregulated p53 pathway (Fig. 7b; Supplementary Fig. 12). The p53 activation is physiologically relevant as seen from the alleviating genetic interaction between *pole1* and *p53* mutations (Supplementary Fig. 3a–d). The overall transcriptional landscape of *dnmt1*/*pole1* double mutants is distinct from the other three genotypes (Fig. 7a), but still largely reflective of the *pole1* mutant-related perturbations (Fig. 7a, b; Supplementary Fig. 12).

Among the 41 differentially expressed genes *dnmt1* single mutants, four patterns of differentially expressed genes can be recognized, when compared to their expression levels in single and double mutants (Fig. 7c, d). Up-regulated (*n* = 19) and down-regulated (*n* = 22) genes split into two clearly discernible groups each. Down-regulated genes in cluster 2 regain wild-type expression levels in double mutants, whereas those for genes in cluster 3 remain low (Fig. 7d). Similarly, expression levels for up-regulated genes in cluster 4 return to baseline, whereas those in cluster 1 stay high (Fig. 7d). This indicates that in the *dnmt1*/*pole1* double mutants, the expression levels of 17 out of 41 dysregulated genes (41%) are restored to normal levels, strongly suggesting that alleviating genetic interactions are not confined to DNA methylation but are also detectable at the transcriptional level. However, CG methylation levels and transcriptional activity for the 41 differentially regulated genes in *dnmt1* single mutants do not correlate (Supplementary Fig. 13a); likewise, no genome-wide correlation was noted between the extent of DNA methylation and transcript levels in the four genotypes of the *dnmt1* and *pole1* comparison (Supplementary Fig. 13b). This finding argues against a straightforward relationship between DNA methylation pattern and gene activity, and rather suggests the presence of complex interactions among the genetic networks regulating gene expression in larvae that develop under conditions of impaired DNA synthesis and post-replicative methylation.

**Fig. 8:**
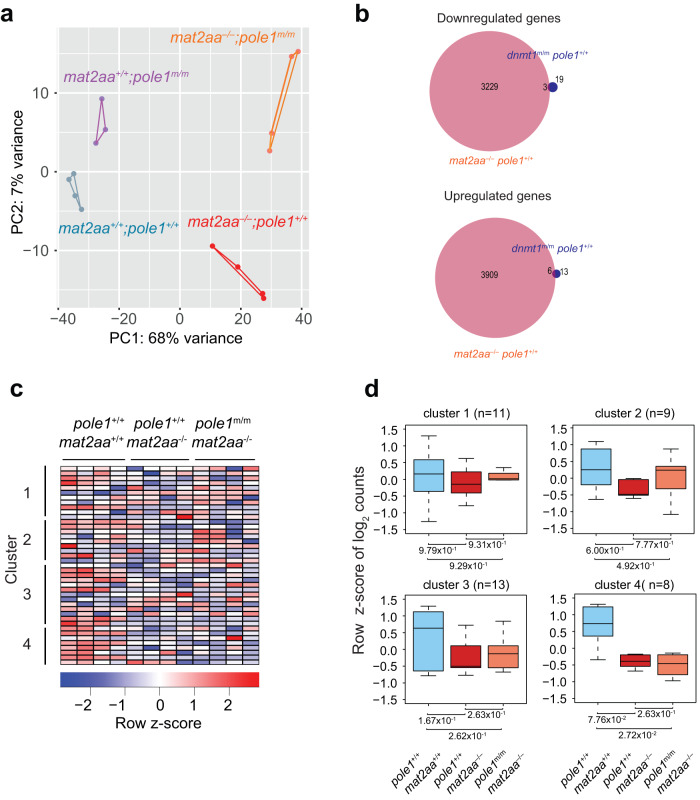
Transcriptional landscapes in *mat2aa* and *pole1* mutants. **a** Dynamic changes in transcriptomes of *mat2aa* and *pole1* single and double mutants, as indicated by principal component analysis. **b** Venn diagram indicating the overlap of differentially expressed genes for down-regulated and up-regulated genes in fish of the indicated genotypes. **c** Expression patterns of genes in the four clusters of co-regulated genes identified in *dnmt1* mutant fish; each column represents a biological replicate. **d** No restoration of expression levels in *mat2aa/pole1* double mutants for the genes in the four groups identified in *dnmt1* mutant fish; box plots (mean; box, upper/lower quartile; whiskers, minimal/maximal values).

Next, we studied transcriptome changes in *mat2aa* single and *mat2aa/pole1* double mutants (Fig. 8; Supplementary Fig. 14). *mat2aa* single mutant larvae are distinguished by more than 7000 differentially regulated genes (Fig. 8a, b; Supplementary Fig. 14), reflecting the profound alterations caused by reduced levels of the essential SAM cofactor. Although the p53 pathway becomes activated in *mat2aa* mutants (Supplementary Fig. 14), the deleterious effect of *mat2aa* deficiency cannot be alleviated by p53 inactivation (Supplementary Fig. 3h), again attesting to a complex cellular response as a result of the *mat2aa* mutation. Of note, only a mere 9 out of 41 genes affected in the *dnmt1* single mutant are also affected in the *mat2aa* single mutant (Fig. 8b), and none of them exhibits changes in expression levels in *mat2aa/pole1* double mutants (Fig. 8c, d). Moreover, when viewed at the global level, the transcriptional dysregulation in *mat2aa* mutants cannot be reversed by the simultaneous presence of the *pole1* mutation (Supplementary Fig. 15), clearly setting this mutant apart from the *dnmt1* mutant-mediated perturbations.

Finally, we examined the transcriptional changes in *mcm10* mutants, focusing on the potential overlap with genes affected in the *pole1* mutation. The milder phenotype of *mcm10* mutants is reflected in less pronounced transcriptional changes when compared to *pole1* mutants; less than 300 genes are differentially expressed in *mcm10* larvae, ten-fold less than in *pole1* larvae (Supplementary Fig. 16a, b). Despite mechanistic links between Pole1 and Mcm10 function, the overlap between the sets of up-regulated and down-regulated genes in these two types of mutants is surprisingly small (Supplementary Fig. 16c), although both mutants share signs of upregulation of the p53 pathway. The up-regulation of the p53 pathway is functionally relevant not only for *pole1* mutants, but also for *mcm10* mutants, since the phenotypic consequences of the *mcm10* mutation are partially ameliorated under conditions of *p53* deficiency (Supplementary Fig. 3e, f).

Collectively, the partial restoration of perturbed transcriptional landscapes in *dnmt1*/*pole1* double mutant larvae is indicative of alleviating genetic interactions between these two genes. This finding highlights the biological relevance of studying hypomorphic rather than null alleles in attempts to define epistatic relationships affecting the outcome of developmental processes.

### Epistatic modulation of mutant phenotypes

**Fig. 9:**
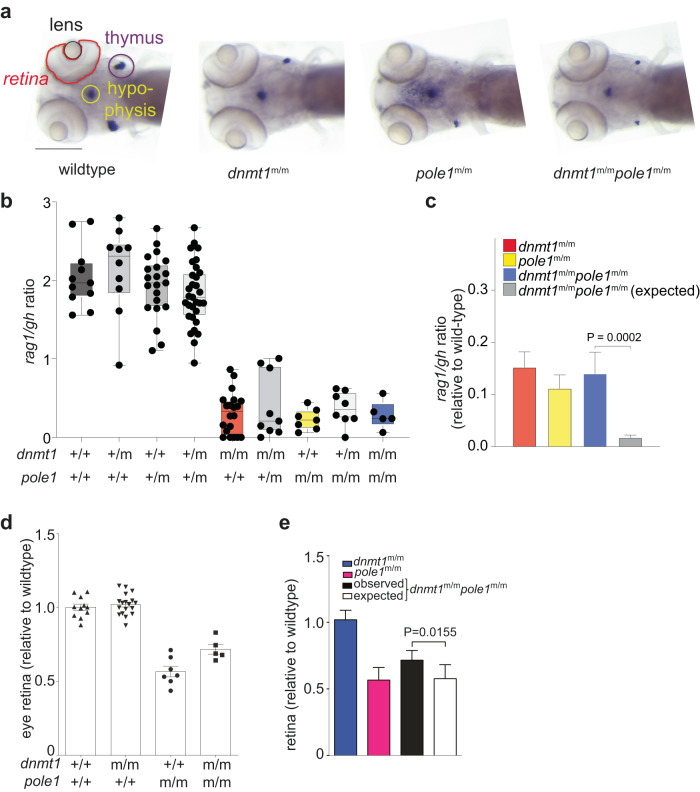
Epistasis analysis of *dnmt1* and *pole1* mutations. **a** Whole-mount RNA in situ hybridization results of larvae of the indicated genotypes are shown (*rag1* signal in thymus [indicated by purple circle]; *gh* signal in hypophysis [indicated by light green circle]). The lens and retina are outlined. Scale bar, 1 mm. **b** Representation of *rag1/gh* ratios of fish with the indicated genotypes (each symbol represents an individual fish); mean±s.e.m. **c** Quantitative analysis of data in (a) (mean±s.d.; t-test; two-tailed); alleviating interaction according to the multiplicative model (see Methods) is demonstrated. **d** Relative size of retinas of fish with the indicated genotypes (each symbol represents an individual fish; mean±s.e.m. **e** Quantitative analysis of data in (d) (mean±s.d.; t-test; two-tailed); alleviating interaction according to the multiplicative model (see Methods) is demonstrated.

Next, we examined possible changes of three selected aspects of mutant phenotypes in the six double mutants examined in this study, covering the development of T cells and the central nervous system. The presence and numbers of developing T cells in the thymus is read out by RNA in situ hybridization with a probe specific for *rag1* (encoding the recombinase required for the assembly of T cell receptor genes in immature thymocytes); the numbers of somatotrophs in the developing hypophysis are determined using a probe specific for the growth hormone gene (*gh*); the development of the retina is approximated by the size of the eye cup (Fig. 9a). Except for the reduced numbers of T cells, *dnmt1* mutants appear to be normal (Fig. 9a), when analyzed at 5 dpf^35,39,40^. By contrast, apart from impaired intrathymic T cell development, *pole1* (Fig. 9a) and *mcm10* mutants (Supplementary Fig. 17) additionally feature a smaller eye-cup, in line with the effects of impaired DNA replication on rapidly proliferating tissues. Remarkably, both types of pathologies are alleviated (but not entirely normalized) in *dnmt1/pole1* double mutants (Fig. 9b–e), whereas the restoration of failing T cell development in *dnmt1/mcm10* double mutants is less pronounced (Supplementary Fig. 17). By contrast, treatment of *pole1* and *mcm10* mutants with 5-aza-2′-deoxycytidine (5azadC), an inhibitor of DNA methylases^55^, does not reverse the mutant-associated pathologies (Supplementary Fig. 18); this failure may be attributed to pan-methylase inhibition, affecting both maintenance and de novo activities. In aggregate, our results suggest that an impaired replication process affords a grace period for the mutant Dnmt1 enzyme to complete (possibly in concert with other methylases) methylation of nascent DNA strands.

**Fig. 10:**
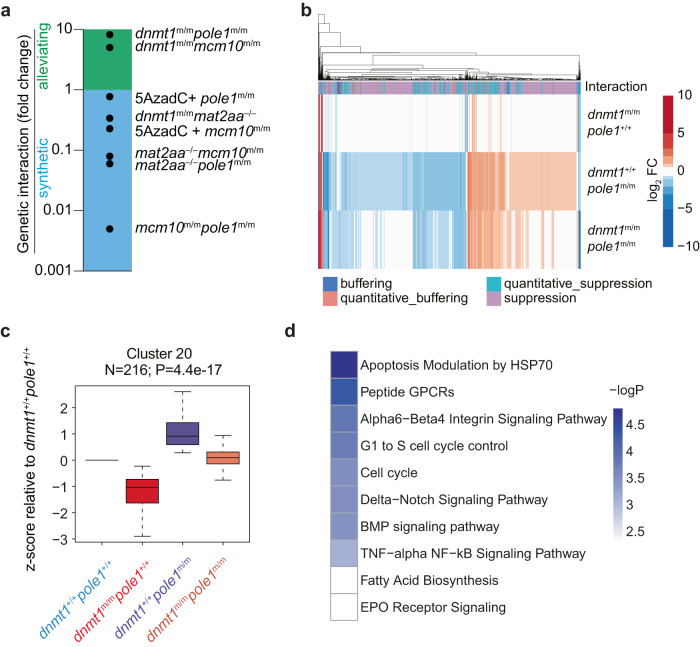
Genetic interaction analysis. **a** Summary of genetic interaction analyses for the indicated pairs using the *rag/gh* ratio as parameter; fold changes are shown relative to the default state (1; no interaction). The multiplicative model was used throughout (see Methods). **b** Global gene expression analysis in the indicated genotypes; the type of genetic interaction follows the nomenclature in^86^. **c** Expression pattern of co-regulated genes in cluster #20 in fish of the indicated genotypes. The numbers of genes per cluster are indicated; box plots (mean; box, upper/lower quartile; whiskers, minimal/maximal values). **d** Pathway analysis of genes in cluster #20 of co-regulated genes (see Supplementary Fig. 23 for full STEM analysis).

As expected, non-compensatory synthetic interactions were observed for *dnmt1/mat2aa*; *mat2aa/pole1*; *mat2aa/mcm10*; and *pole1/mcm10* double mutants, whereas some alleviation was observed in the *dnmt1/mcm10* double mutant (Supplementary Figs. 17, 19–22). The outcomes of alleviating and synthetic genetic interactions for the different genetic and pharmacological conditions are summarized in Fig. 10a.

Given the strong epistatic interactions, we comparatively re-evaluated the global differences in transcriptional landscapes in *dnmt1* and *pole1* single mutants, and *dnmt1/pole1 double* mutants, highlighting pervasive compensatory effects of the *pole1* mutation at the level of individual transcripts (Fig. 10b). Using a trend-mining algorithm^56^, several groups of genes exhibiting coordinated changes can be distinguished (Supplementary Fig. 23). For instance, pattern #20, comprising about 200 genes, is categorized as representing a typical suppressive/alleviating interaction mode; the opposing deregulations caused by the two single mutants are canceled out in the double mutant to reestablish wild-type levels (Fig. 10c). Interestingly, this group of genes is enriched for cell cycle-related genes as well as for those encoding components of the Delta-Notch and BMP signaling pathways (Fig. 10d), which are known to be important for T cell^57,58^, and craniofacial^59^ development. The restoration of expression levels of these genes may thus collectively contribute to the less severe phenotype of *dnmt1*/*pole1* double mutants.

## Discussion

Our study illuminates the functional link between DNA replication and DNA methylation in a developing vertebrate organism. When cell divisions occur in rapid succession, as is the case during the early stages of zebrafish development, efficient post-replicative methylation is critical to re-establish the parental methylation patterns in daughter cells before the next mitosis commences^60^.

Our results indicate that global CG methylation levels are very sensitive to impaired enzymatic activity of Dnmt1 and also shed light on the role of Dnmt1 during establishment and/or maintenance of non-CG methylation. The clearest difference between CG and non-CG methylation emerges with respect to the overall loss of methylated sites in the genome of *dnmt1* mutants. Whereas this loss is negligible for CG dinucleotides, it is much more pronounced at CHG and CHH sites. However, the reduction in the mean methylation ratios of the remaining sites is reduced to approximately similar extents for the three classes. Another apparent distinction is seen with respect to the aberrant hypermethylation in *pole1* mutants. Again, whereas mean methylation ratios modestly increase at CG dinucleotides without recruitment of many new sites, more profound changes occur at CHG and CHH sites: More sites become methylated, accompanied by an overall increase of mean methylation ratios. Overall, our results indicate that impaired enzymatic activity of Dnmt1 causes the loss methylation in all three sequence contexts studied here. These findings are compatible with the notion that non-CG methylation occurs in regions with high density of CG methylation^12^. It should be noted however that the specific missense mutation encoded by the mutant *dnmt1* allele has particular consequences that may differ from those of other aberrations in the Dnmt1 enzyme, such as those giving rise to neurological disorders^61^. More work will be required to explore the possibility that Dnmt1 exhibits increased de novo activity when the replication process itself is impaired, and to establish the phenotype of additional mutations in individual members of the Dnmt3 family of enzymes.

Although our results suggests that impaired Dnmt1 activity cannot be fully compensated by other DNA methylases of the Dnmt3 family, it is nonetheless surprising that the hypomorphic allele of *dnmt1* does not impair viability and fertility^35^. Given the lethal phenotype of *dnmt1* null alleles^37,38^, we interpret this finding to indicate that the mutant-associated mean methylation ratios are still above the critical threshold level that would be associated with early lethality.

Another interesting aspect of the phenotype of *dnmt1* mutants concerns the relationship between overall levels of DNA methylation and transcriptional activity. Although it has been proposed that methylation of gene bodies serves to suppress aberrant initiation of transcription^62^, the widespread demethylation associated with impaired activity of Dnmt1 does not cause dramatic changes in the transcriptional landscape. By contrast, impaired Pole1 activity causes a dramatic remodeling of the transcriptome. Considering the observation that impaired replication increases cytosine methylation ratios, we propose that at least part of the complex developmental abnormalities and increased tumor susceptibility associated with Pole1 hypomorphy^49,63–65^ are caused by aberrant hypermethylation of DNA. The antagonistic interaction between DNA replication and DNA methylation identified here in an in vivo vertebrate model system offers the prospect of interfering with the phenotypic consequences of this syndrome.

## Methods

### Animals (zebrafish)

The zebrafish (*D. rerio*) wild-type strain TLEK (Tüpfel long fin/Ekkwill) is maintained in the animal facility of the Max Planck Institute of Immunobiology and Epigenetics, Freiburg, Germany and was used for crosses with the *dnmt1*, *pole1*, *mcm10*, and *mat2aa* mutants^39,40^. The *ikzf1:EGFP* transgenic reporter line^46^ and the *p53* mutant M214K (Ref ^50^.) were used; the p53(p.M214K) strain (established in the laboratory of A. T. Look) was obtained from M. Hammerschmidt. Zebrafish of different ages were used, without regard to sex. All zebrafish experiments were performed in accordance with relevant guidelines and regulations, approved by the review committee of the Max Planck Institute of Immunobiology and Epigenetics and the Regierungspräsidium Freiburg, Germany (license 35-9185.81/G-14/106; license 35-9185.81/G-15/115).

### Identification of a hypomorphic allele of zebrafish *pole1*

For fine-scale mapping, new markers were generated for chromosome 5: 13B6_1 (5´-ggtgaaagaagcctggttttca and 5´-ccggaagctgaaattagtagac [nt 13,809,479 to 13,809,500 and 13,809,772 to 13,809,793]), 98C11_1 (5´-aactcacgccaaatggaaag and 5´-taaacgccgcagtctttagg [nt 13,896,113 to 13,896,132 and 13,895,973 to 13,895,992]), 86g2_tg1 (5´-tgatgaaacctctgcgactg and 5´-catcttctccctcacctcca [nt 14,056,181 to 14,056,200 and 14,056,299 to 14,056318]); coordinates are from Zv9 (April 2010; database version 66.9) at http://www.ensembl.org/Danio_rerio/Info/Index. The critical region is flanked by markers 98C11_1 (genetic distance from mutant locus < 0.2 cM) and 86g2_tg1 (genetic distance from mutant locus < 0.1 cM) (Fig. 2c) and is covered by four BAC clones (CH73-13B6, Dkeyp-98C11, CH73-135P21 and Dkeyp-86g2). Note that the wildtype sequence for the relevant isoleucine codon in the genomic DNA of the Tübingen strain is ATA instead of ATC in the reference sequence (Genbank accession number NM_001128523); hence, the mutation thus changes the ATA codon to AAA. The following primers were used for genotyping: forward primer, 5´-gtctgtggacatttgatgcttg; reverse primer, 5´-gactccagcttggacccac; amplicons were directly sequenced with the reverse primer. For sequence analyses, fish were sorted based on phenotype after *rag1/gh* whole-mount RNA in situ hybridization; sequences were carried out on mutant and wildtype DNA pools. In the BAC rescue experiment (see Fig. 2), the craniofacial phenotype was normalized in 10 out of 22 mutant embryos, as determined by alcian blue staining.

### Identification of a hypomorphic allele of zebrafish *mcm10*

For fine-scale mapping, two additional markers were developed. IG335_125 (forward primer, 5′-gtcccgaacacccactaacat; reverse primer, 5′-ggtctgaccttccactaacac) and IG335_200 (forward primer, 5′-acgttttcaggcctactatgtc; reverse primer, 5′-cgtggcattaaaggcttgctg). Detailed analyses of genotypes in the mapping crosses identified a critical interval of about 200 kb in chromosome 4 containing six candidate genes; the critical interval extends across 0.125 cM and the mutation was found to be 0.04 cM away from the closest informative marker, IG335_125 (Supplementary Fig. 4a). All genes in the critical interval were completely sequenced, including splice donor and acceptor sites from IG335 mutant and wildtype DNA pools (fish were sorted based on phenotype after *rag1/gh* whole-mount RNA in situ hybridization). For genotyping, the following primers were used: Forward primer, 5´-aggaagccacgtctgtcttc; reverse primer, 5´-gatctcagatatgctgctcca; amplicons were directly sequenced with the reverse primer. In *mcm10* morphants, the morpholino-induced impaired pre-mRNA splicing of *mcm10* mRNA was confirmed by RT-PCR.

### Characterization of a hypomorphic allele of zebrafish *dnmt1*

A detailed characterization of the *dnmt1* mutant (allele designation t25501) has been given elsewhere^35,36^. Note that the *dnmt1* phenotype is not affected by the lack of p53 activity (Supplementary Fig. 3g). The following primers were used for genotyping: Forward primer, 5′- ctgagaggagtgtgttcatgt; reverse primer, 5′- ctggtcagcatatggcatgta; amplicons were directly sequenced with the reverse primer.

### Identification of a nonsense mutation in the zebrafish *mat2aa* gene

The *mat2aa* mutant (allele designation t24600) has been described elsewhere^39,40^. Note that the *mat2aa* phenotype is not affected by the lack of p53 activity (Supplementary Fig. 3h). The following primers were used for genotyping: Forward primer, 5′- cccaactaaccaagccaagtt; reverse primer, 5′- agtctcgtcagtggcataac; amplicons were directly sequenced with the reverse primer.

### Animals (mouse)

Mice were kept in the animal facility of the Max Planck Institute of Immunobiology and Epigenetics under specific pathogen-free conditions. Mice were used at different ages, as indicated, without regard to sex. All mouse experiments were performed in accordance with the relevant guidelines and regulations, approved by the review committee of the Max Planck Institute of Immunobiology and Epigenetics and the Regierungspräsidium Freiburg, Germany (license AZ 35-9185.81/G-15/35; license AZ 35-9185.81/G-15/80; license AZ 35-9185.81/G-17/03).

### Mouse lines

Mouse *Pole1* mutants (I634K; S643H; and the equivalent of the human g.G4441 + 3 A > G splice site mutation) were generated by CRISPR-Cas9 technology and locus-specific sgRNAs. Sequence-specific single-stranded repair oligonucleotides contained additional mismatches to avoid cleavage by the pre-assembled sgRNA/Cas9 RNP. The targeting sequences for guide RNAs were designed according to published instructions^66^ and cloned into the pDR274 vector (Addgene plasmid #42250). After digestion with *Dra*I restriction enzyme (New England Biolabs), sgRNA was generated by in vitro transcription using MAXIscript T7 Transcription Kit (Thermo). For injection of zygotes into both the pronucleus and the cytoplasm, two sgRNAs per target site were combined (final concentration 25 ng/μL), recombinant Cas9 protein from *Streptococcus pyogenes* (PNA Bio; final concentration 50 ng/μL), and repair oligonucleotide (final concentration 5 μM) were mixed on ice in 10 mM Tris.HCl, pH 7.5; 0.15 mM EDTA; approximately 1–2 nL of the solution were injected per fertilized egg. The following guide sequences were used to introduce double-strand breaks: I634K mutation, 5′-cctaacataattcttaccaa; S643H, 5′-ttctgccatagtggatgagg; splice site mutation, 5′-ggctgttggtaaggcaatac. The following primer pairs were used to determine the respective genotypes: I634K and S643H mutations, forward 5′-ctttgtaggtgtgtgagcag, reverse 5′-ggccatcttcctctgacaac; splice site mutation, forward 5′-gctgggaagctgaaacttttg, reverse 5′-cagatgctcttcgctgtgag. The amplicons were sequenced with one of the amplification primers. The following single-stranded repair oligonucleotides were used: I634K mutation, 5′-gagaagtgactatgatatgagaagggaggtcttacctgtaggcggttggtcaaaattttgttaggatacatggcccccacatctagatggtagattagaggacattca;

S643H mutation, 5′-ctctttgcaggcccaagttgatttctaaccctcccctcctttcctagccacatgccatagtggatgaggccacctgtgctgcctgtgacttcaataagcc;

splice site mutation, 5′-gcaccttgaaatgcgttctctggcccagttcagctacttggaaccaggtgtggccaaaccaacagccctatcacctaccttgcctcccctgctctcaggc. The I634K mutation was introduced first (B6N;FVB/N-*Pole1*^em1Tbo^/Mpie), followed by the S643H mutation (B6N;FVB/N-*Pole1*^em2Tbo^/Mpie); double-mutated mice (B6N;FVB/N-*Pole1*^em3Tbo^/Mpie) were crossed to wild-type mice to check for co-transmission of the two missense mutations on the same allele.

### Zebrafish morphants

Morphants were generated by injection of anti-sense morpholino oligonucleotides (Gene Tools, Philomath, OR) to block translation of both maternal and zygotic mRNAs (ATG morpholinos), or to block splicing of zygotic pre-mRNAs (“splice morpholinos”). Stock solutions of morpholinos were diluted as required in injection buffer (final concentration, 0.05% (v/v) phenol red at concentrations of 0.1 mM, 0.2 mM, or 0.5 mM; 1x Danieau Buffer (http://cshprotocols.cshlp.org/content/2011/7/pdb.rec12467.full). Approximately 1–2 nL of solution were injected into fertilized eggs at the one-cell stage. The following morpholinos were used. *pole1*, 5′-gtctgaagactttcaaatcagttac (encompassing the sequences of the initiation codon). *mcm10*, 5′-tctgaagaggctgatttacataaga (blocking the exon 4 splice acceptor site). The sequences of the anti-p53 oligonucleotide (5′-agaattgattttgccgacctcctct) and of the anti-*pold1* oligonucleotide (5´-ccatttttaagcagctcttttaatc) were taken from^48^.

### mRNA and BAC injections in zebrafish

BAC DNAs or mRNAs were injected into zebrafish embryos resulting from intercrosses of heterozygous parents at the one-cell stage. BAC DNAs were injected at concentrations of 50 ng/μl; mRNAs were injected at concentrations of 100 ng/μl. Phenotypic rescue of the *pole1* mutants was carried by injection of a mouse BAC clone encompassing the *Pole1* gene (RP23-408F15 [RPCIB731f15408q]; Imagenes, Berlin, Germany). The rescue of *mcm10* mutants was attempted by injections of mouse *Mcm10* mRNA (Genbank accession number BC120687; clone 40129610) and a mouse BAC clone containing the wildtype *Mcm10* gene (Genbank accession number AL928662), respectively. The injected larvae were analyzed at 5 dpf by RNA in situ hybridization using a combination of *rag1*- and *gh*-specific probes, and the results expressed as a thymopoietic index, a dimensionless number (see below).

### Thymopoietic index in zebrafish larvae

In the present work, the intensity of the RNA in situ signal of *rag1* is considered to be a surrogate measure of the number of differentiating T cells, which we consider to be a measure of T cell development. As an internal control (technical, with respect to the hybridization process as such; and, biological, with respect to the tissue specificity of the observed genetic effects), a probe specific for the growth hormone (*gh*) gene, which marks a subset of cells in the hypophysis, is used. To determine *rag1/gh* ratios, the following procedure was carried out after RNA in situ hybridization with *rag1* and *gh* probes. Ventral images of 4–5 dpf zebrafish larvae were taken on an MZFLIII (Leica) microscope using a digital camera DFC300FX (Leica), essentially generating a two-dimensional projection of the three-dimensional structure. The areas of *rag1* and *gh* signals were measured using ImageJ (ImageJ 1.52a; available at http://imagej.nih.gov/ij), and the ratio of average of the *rag1*-positive area vs. *gh*-positive area was calculated. This represents a measure of thymopoietic activity. Larvae were processed for genomic DNA extraction and subsequent genotyping after photographic documentation of the RNA in situ hybridization signal.

### Genetic interaction determination and nomenclature

T cell and retinal fitness values (*W*) for single mutants (*W*_x_ and *W*_y_) and double mutants (*W*_xy_) were calculated as follows. First, *rag1*/*gh* values and eye dimensions, respectively, were normalized to the corresponding wild-type values. To calculate expected fitness E(*W*_xy_)=*W*_x_ * *W*_y_, the multiplicative model was chosen as it was the most accurate model in predicting observed fitness as determined by the residual mean squared error^40^. To calculate the propagated error for the expected fitness ε_E(Wxy)_ standard deviations δ_Wx_ and δ_Wy_ and fitness values *W*_x_ and *W*_y_ of the single mutants were used in the following equation, ε_E(Wxy)_= [(δ_Wx_ /*W*_x_)^2^ + (δ_Wy_ /*W*_y_)^2^]^−2^. The degree of genetic interaction was determined as the log_2_ fold-change between observed *W*_xy_ and expected E(*W*_xy_) fitness values.

### RNA extraction and cDNA synthesis

The manufacturerʼs instructions were followed for the extraction of total RNA using TRI Reagent (Sigma). After treatment with DNaseI (Promega), RNA was re-extracted using TRI Reagent. Superscript II Reverse Transcriptase (Invitrogen) and oligo(dT) were used for cDNA synthesis from total RNA.

### Quantitative PCR

qPCR was carried out using SYBR Premix Ex Taq (Takara) and 7500 fast real-time PCR system (Applied Biosystems). *actb1* was used as a reference gene. The primer sets for zebrafish genes were purchased from BioRad (https://www.bio-rad.com/de-de/product/primepcr-pcr-primers-assays-arrays?ID=M0HROA15). *dnmt1*, qDreCED0019976; *dnmt3aa*, qDreCID0018392; *dnmt3ab*, qDreCID0019082; *dnmt3ba* (aka *dnmt3b*), qDreCED0021338; *dnmt3bb.1* (aka *dnmt4*), qDreCID0005035; *dnmt3bb.2* (aka *dnmt3*), qDreCID0016654; *dnmt3bb.3* (aka *dnmt5*), qDreCED0021863; *tet1*, qDreCED0015074; *tet2*, qDreCED0010969; *tet3*, qDreCID0016164; *actb1*, qDreCED0020462.

### Histological analysis

For histological analysis, specimens were fixed in formalin, the embedded in paraffin; sections were stained with hematoxylin/eosin. For image analysis of histological sections, JmageJ software was used (ImageJ 1.52a; available at http://imagej.nih.gov/ij).

### Imaging of zebrafish specimens

Embryos and larvae were anesthetized and immobilized in 3% methylcellulose. Fluorescence microscopy was performed with Zeiss Imager.Z1.

### Whole-Mount RNA in situ hybridization

Digoxigenin-labeled RNA riboprobes^40,45^ were employed for whole-mount RNA in situ hybridization of zebrafish larvae.

### Alcian blue staining

Cartilage structures were visualized by alcian blue staining^40,45^.

### Treatment of zebrafish embryos with Dnmt inhibitors

A stock solution of 5-Aza-2′-deoxycytidine (5AzadC; Sigma) was prepared in E3 medium, and diluted to the desired final concentration of 5 μM. Wild-type embryos were exposed to the inhibitor^67^ beginning at 24 hpf for a total of 48 h; at 72 hpf, embryos were washed and continuously cultured in E3 medium.

### Timed matings of mice

Timed matings were used to retrieve embryos at different time points; the day of the plug was counted as day E0.5 of gestation. Zygotes were cultured in vitro in KSOM media (Cosmobio) drops under Ovoil culture oil (Vitolife) until the morula and blastocyst stages.

### Yeast procedures

A linearized integration plasmid^68^ carrying mutations resulting in a I648K variant or a I648K, D657H variant was integrated into a diploid E134 yeast strain (*MATα/MATa ade5-1/ade5-1 lys2::InsEA14/lys2::InsEA14 trp1-289/trp1-289 his7-2/his7-2 leu2-3,112/leu2-3,112 ura3-52/ura3-52*). URA3 was used for the selection of integration. Four integrants from each transformation were isolated and then patched on YPD overnight to allow for the looping out of the URA3 marker, leaving the specific *pol2* mutation on the chromosome. Patched clones were then replica-plated on 5-FOA plates to select for clones that had lost URA3. Three 5-FOA^r^ clones from each patch were picked and streaked for single colonies on YPD. PCR was used to screen colonies for the desired mutation and positive diploid clones were sequenced across the *POL2* gene to confirm that the selected mutation was correctly integrated in the heterozygote strain and to verify that there were no additional mutations. At least 8 tetrads from each strain were dissected^69^ and the haploid cells were from this point grown at 22 °C. When plating the I648K strain on YPD plates that were initially incubated at 30 °C some larger colonies were observed on the plate. After sequencing the *POL2* gene in these isolates we found a new variant – D657H – that was confirmed to partially suppress the temperature-sensitive phenotype of the I648K strain. Two isolates of each strain, E134 wildtype strain; I648K mutant; and I648K;D657H double-mutant, respectively, were grown on YPD plates at 22 °C. Single colonies from these plates were streaked on fresh YPD plates that were incubated at 22 °C or 37 °C, respectively, for up to 6 days. To obtain the growth curve, 15 ml overnight starter cultures (YPD medium at 22 °C) were used to inoculate 100 ml fresh YPD medium. The cultures were grown at 22 °C and 37 °C, respectively, and the OD was monitored at A_600_ nm.

### RNA-Seq library preparation

Total RNA was extracted from whole zebrafish larvae at 5 dpf using TRI Reagent (Sigma) following the manufacturer’s instructions. After treatment with Cloned DNaseI (Takara), RNA extraction using TRI Reagent was repeated. Libraries were prepared via the TruSeq® mRNA library preparation protocol according to manufacturer’s instructions. Libraries were sequenced on an Illumina HiSeq 2500 according to manufacturer’s instructions.

### Bisulfite-sequencing

Genomic DNA was extracted from 5dpf zebrafish embryos using the DNeasy blood and tissue kit (Qiagen). 0.5 µg of DNA was used for bisulfite reactions and library construction using the EpiGnome Methyl-Seq kit (Epicentre). Libraries were sequenced on an Illumina HiSeq 2500 according to manufacturer’s instructions.

### RNA-Seq processing

RNA-Seq processing was performed using snakePipes 2.4.2 (Ref. ^70^). Reads were aligned to the GRCz11 genome using STAR version 2.4.7a (Ref. ^71^) using --outStd BAM_Unsorted --outSAMtype BAM Unsorted --outSAMunmapped Within--sjdbGTFfile --sjdbOverhang 100 as parameters. Mapped reads were weeded for PCR duplicates using sambamba version 1.0.0 (Ref. ^72^) using --sort-buffer-size=6000 --overflow-list -size 600000 as a parameter. Counts were obtained using featureCounts from the subread package version 2.0.0 (Ref. ^73^) using -p -B -C -Q 10 --primary -T 8 -s 2 -a as parameters. Read coverages were obtained using deeptools bamCoverage version 3.3.2 (Ref. ^74^), with --binSize 25 --normalizeRPKM --effectiveGenomeSize 1679186873 --maxFragmentLength 1000 --scaleFactor as parameters following deeptools multiBamSummary version 3.3.2 using bins --scalingFactors as parameters. Differential gene expression analysis: differentially expressed genes were obtained DESEq2 version 1.26.0 (Ref. ^75^) using alpha = 0.05 as a parameter. Final normalized, r-log transformed count values were obtained using the rlog function. For clustering analyses, row z-scores of rlog transformed data for *dnmt1*^+/+^/*pole1*^+/+^; *dnmt1*^m/m^/*pole1*^+/+^ and *dnmt1*^m/m^/*pole1*^m/m^ datasets for all 41 differentially regulated genes were used. k-means clustering was performed using the kmeans R function^76^. The Short Time-series Expression Miner (STEM) algorithm was used for the clustering of co-regulated genes^56^.

### Gene ontology analysis

The analyses were performed using Homer findgo.pl (Ref. ^77^) with -bg zebrafish.base.gene as a parameter.

### WGBS processing

WGBS processing was performed using snakePipes 1.2.3 (Ref. ^70^). Reads were adapter-, quality- and end- trimmed using cutadapt 2.1 (Ref. ^78^) using -a AGATCGGAAGAGC -A AGATCGGAAGAGC -q 10 -m 30 -j 8 as parameters. Reads were aligned to the bisulfite-converted GRCz11 genome using bwa-meth version 0.2.2 (Ref. ^79^) using --read-group as a parameter. Mapped reads were weeded for PCR duplicates using sambamba version 0.6.6 using --remove-duplicates as a parameter. CpG methylation extraction and coverages were obtained using methyl_extract version 1.9 (Ref. ^80^) using default parameters. Differential methylation analysis was performed using metilene 0.2.6 (Ref. ^81^) with default parameters. Average coverage values were computed using the bedtools merge function (Ref. ^82^) with -c 4 -o mean as parameters on bedGraph files obtained from the bigWigToBedGraph function (Ref. ^83^). Spearman correlation clustering was performed using deeptools multiBigWigSummary bins function followed by plotCorrelation using --corMethod spearman and --removeOutliers as parameters. Average methylation profiles were generated using deeptools computeMatrix using --referencePoint TSS -b 3000 -a 3000 as parameters, followed by plotProfile.

Differentially-methylated regions (DMRs) were computed via metilene using the following parameters: maxDist: 300 minCpGs: 10 minCoverage: 5 FDR: 0.1 minMethDiff: 0.1. Note that our analysis cannot distinguish between hemimethylated and fully methylated cytosine residues.

For pair-wise comparisons, methylation coverages and ratios were computed from CpG positions in the reference genome with a minimum coverage specified by --minCoverage and low SNP allelic frequency ( < 0.25 illegitimate bases) in both conditions.

### Comparison of CpG, CHG and CHH methylation levels

MethylDackel CpG, CHG and CHH methylation coverage files were obtained via snakePipes 2.4.2 (Ref. ^70^) using WGBS --DAG -j 10 -i <FASTQ > -o <output_folder> GRCz11.yaml --trim --MethylDackelOptions “--CHH --CHG --mergeContext --maxVariantFrac 0.25 --minDepth 4”. To reduce skewing in violin plots due to high numbers of 0 methylation sites in CHG and CHH coverages, CpG, CHG and CHH coverage files were then filtered against 0, covered values. In the case of the *mat2aa/pole1* CHH triplet, stringency was increased to values less than 25% methylation to account for very high numbers of fixed value artefacts between 0 and 25%. CpG, CHG and CHH coverage files were then sequentially processed in snakePipes 1.2.3 as methXT files and in the case of CHG and CHH files, renamed as “CpG” to produce violin plots for each condition group using WGBS --DAG -j 10 -i <FASTQ > -o <output_folder> GRCz11.yaml --sampleSheet <samplesheet > . The number of CpG, CHG and CHH sites numbers was derived by obtaining the union of all sites for all replicates per condition via bedtools multiinter -i <all_replicate_coverage_files_per_condition> | wc -l. Median and m.a.d. values were derived in R using apply(metilene.IN, 2, “<mean | mad > ” from intermediary “merged_methylation_data_XXX/metilene.IN.txt” files representing the intersection of coverages for all replicates in all conditions per group, i.e., that shown in violin plots.

### Gene set enrichment analysis

Gene set enrichment analyses were performed using GSEA version 4.0.3 (Ref. ^84^) with gene_set permutation type, weighted enrichment statistic, Diff_of_classes as the ranking metric on log2 transformed gene expression data.

### Statistics and reproducibility

No randomization of animals was done in the present studies; phenotypes were recorded by a blinded observer before genotyping. No animals were excluded from analyses. t-tests were performed for samples with equal variance; otherwise, F-tests were used. The minimum number of biological replicates used for statistical analysis was three, but often included many more, as indicated in the Source Data files.

### Reporting summary

Further information on research design is available in the Nature Portfolio Reporting Summary linked to this article.

### Supplementary information

Supplementary Information

Description of Additional Supplementary Files

Supplementary Data 1

Reporting Summary

## Data Availability

RNA-Seq and WGBS data have been deposited at the Gene Expression Omnibus (GEO) under accessions GSE181571 and GSE181572, respectively. The source data used to generate Figs. 3b, 4, 5a, b, 6a, c, 7b, d, 8b, d, 9b–e, 10a–d; Supplementary Figs. 1a–f, 1f, 2b–e, 3a, 3–h, 4e, 10b, 12a–e, 14a–e, 15, 16a–c, 17b–c, 18a–d, 19b, c, 20b, c, 21b, c, 22b, 22c, 23b are provided in Supplementary Data 1. The data files used to generate Figs. 3a, b, 5b, 6b; and Supplementary Figs. 5a–d, 6a–d, 7a–d, 8a–d, 10b are deposited in the Figshare database under 10.6084/m9.figshare.24711120 (ref.^85^).
