# Author Correction: Antagonistic interactions safeguard mitotic propagation of genetic and epigenetic information in zebrafish

**DOI:** 10.1038/s42003-024-05899-y

**Published:** 2024-02-29

**Authors:** Divine-Fondzenyuy Lawir, Cristian Soza-Ried, Norimasa Iwanami, Iliana Siamishi, Göran O. Bylund, Connor O´Meara, Katarzyna Sikora, Benoît Kanzler, Erik Johansson, Michael Schorpp, Pierre Cauchy, Thomas Boehm

**Affiliations:** 1 Department of Developmental Immunology, Max Planck Institute of Immunobiology and Epigenetics, Freiburg, Germany; 2 Department of Medical Biochemistry and Biophysics, Umeå University, Umeå, Sweden; 3 Bioinformatic Unit, Max Planck Institute of Immunobiology and Epigenetics, Freiburg, Germany; 4 Transgenic Mouse Core Facility, Max Planck Institute of Immunobiology and Epigenetics, Freiburg, Germany; 5 Institute for Immunodeficiency, Center for Chronic Immunodeficiency (CCI), University Medical Center, Faculty of Medicine, University of Freiburg, Freiburg, Germany

**Keywords:** Developmental biology, Genetics, Immunology

Correction to: *Communications Biology* 10.1038/s42003-023-05692-3, published online 5 January 2024

In the original version of this Article, the x-axis labels for *dnmt1*^*m/m*^*/pole*^*+/+*^ and *dnmt1*^*+/+*^*/pole*^*m/m*^ genotypes were mistakenly inverted in Figure 3a (leftmost panel) and 5b.

The original Fig 3a:
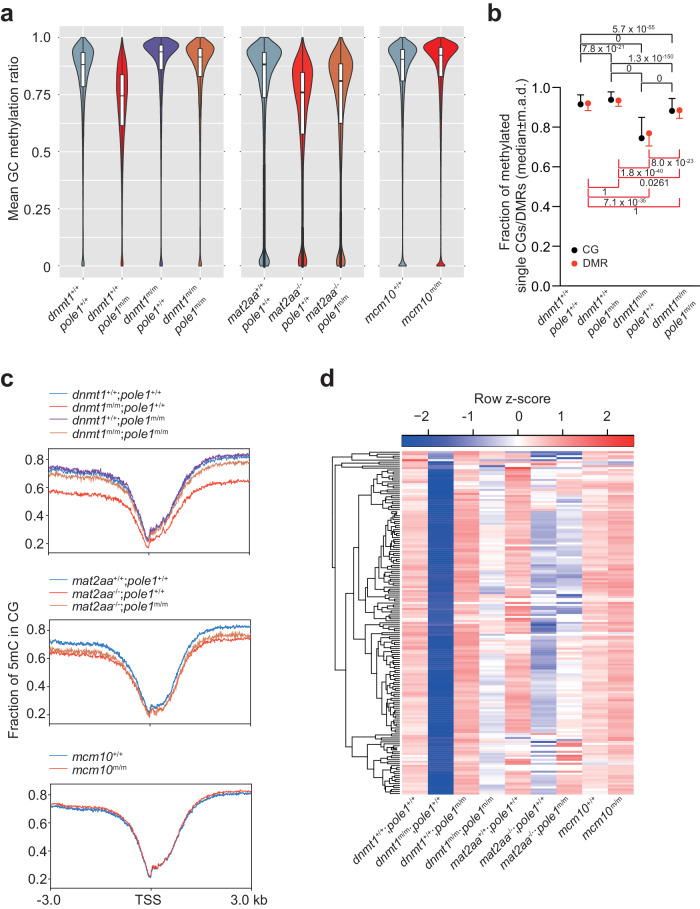


Has now been corrected:
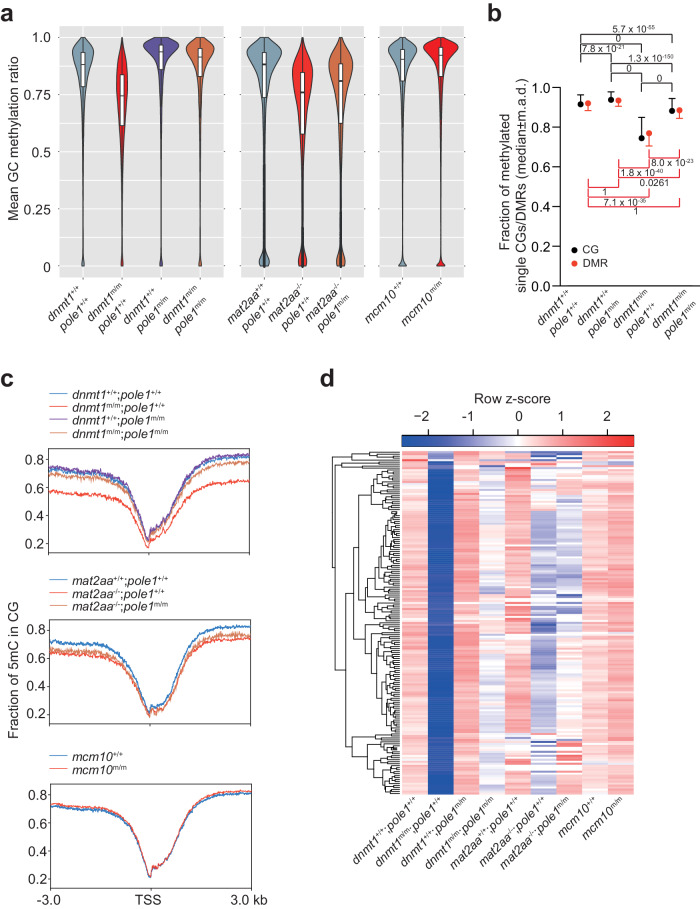


And the original Fig 5b:
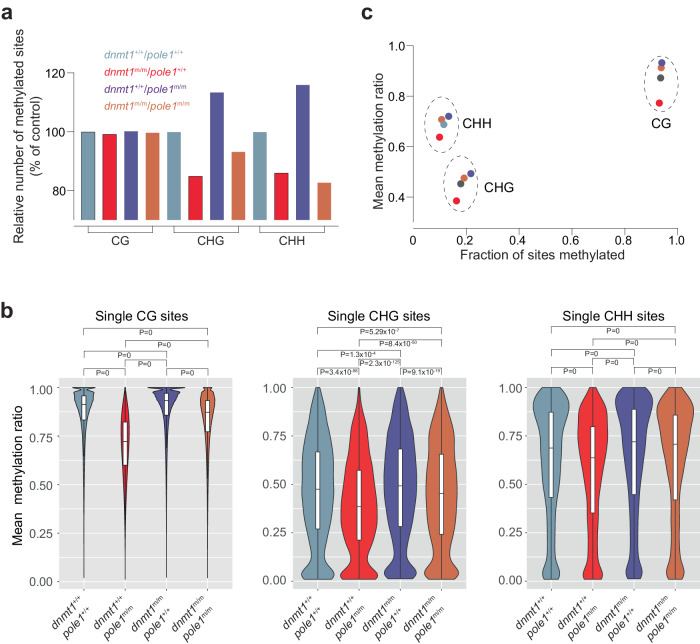


Has also been corrected:
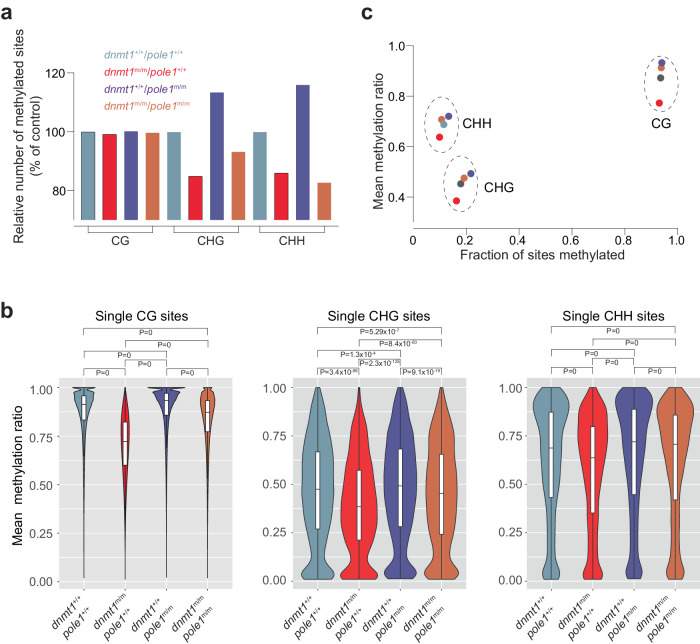


These changes are now reflected in both the PDF and HTML versions of the article.

