# Supplementary material for: Antagonistic interactions safeguard mitotic propagation of genetic and epigenetic information in zebrafish
Source: Commun Biol. 2024 Jan 5;7:31. doi: 10.1038/s42003-023-05692-3 (PMC10770094; doi:10.1038/s42003-023-05692-3)
Supplement: Supplementary file 1 — Supplementary Information [file 42003_2023_5692_MOESM1_ESM.pdf]

Supplementary Information

for

Antagonistic interactions safeguard mitotic propagation of genetic and epigenetic information in zebrafish

Divine-Fondzenyuy Lawir, Cristian Soza-Ried, Norimasa Iwanami, Iliana Siamishi, Göran O. Bylund, Connor O´Meara, Katarzyna Sikora, Benoît Kanzler, Erik Johansson, Michael Schorpp, Pierre Cauchy & Thomas Boehm

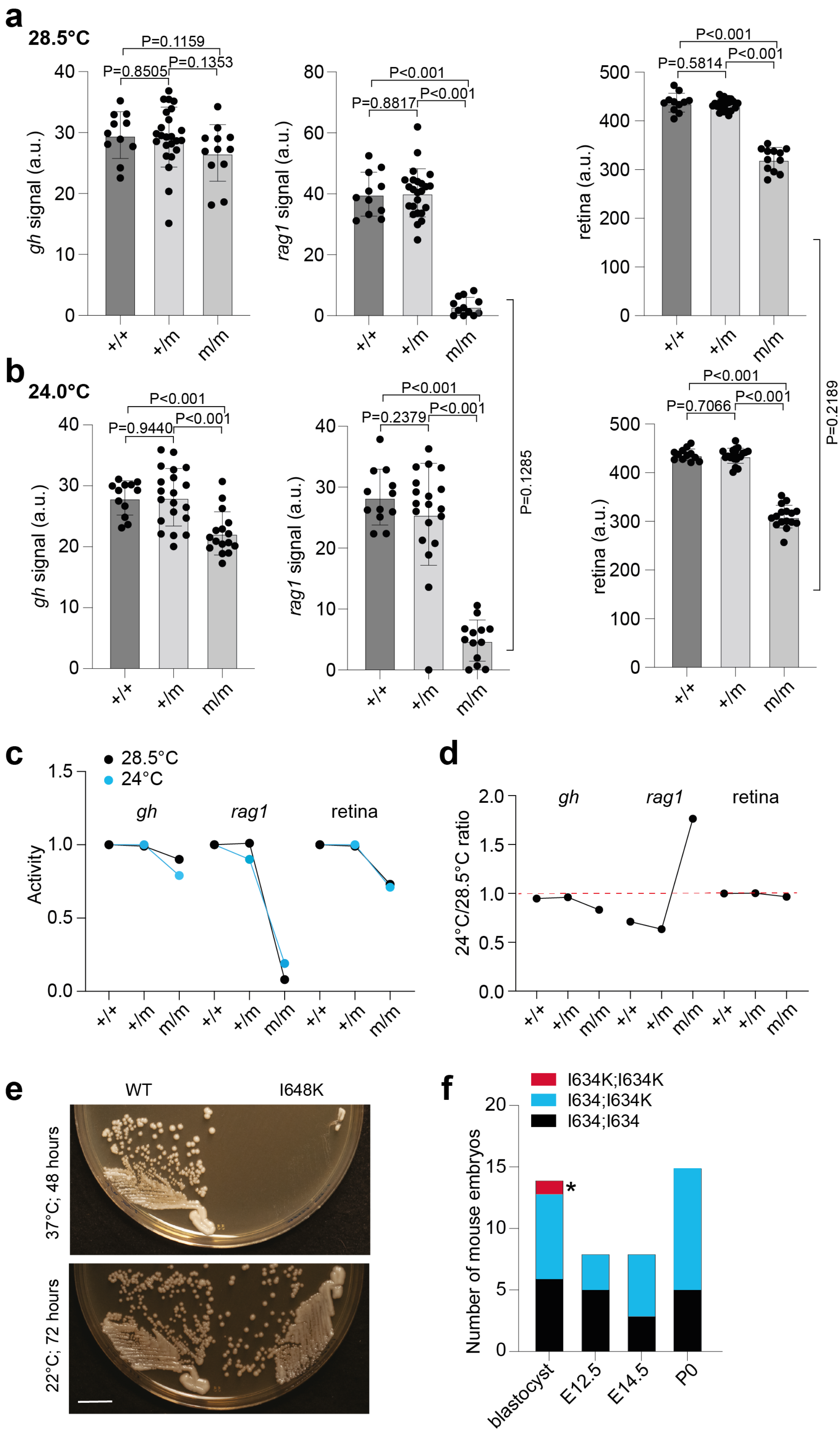
a
28.5°C
gh signal (a.u.)
rag1 signal (a.u.)
retina (a.u.)
P=0.1159
P=0.8505
P=0.1353
P<0.001
P=0.8817
P<0.001
P<0.001
P=0.5814
P<0.001
+/+
+/m
m/m
b
24.0°C
P<0.001
P=0.9440
P<0.001
P<0.001
P=0.2379
P<0.001
P<0.001
P=0.7066
P<0.001
P=0.1285
P=0.2189
c
28.5°C
24°C
Activity
gh
rag1
retina
d
24°C/28.5°C ratio
e
WT
I648K
37°C; 48 hours
22°C; 72 hours
f
I634K;I634K
I634;I634K
I634;I634
Number of mouse embryos
blastocyst
E12.5
E14.5
P0

Supplementary Figure 1. The pole1 (p.I633K) variant is temperature sensitive. a *pole1*$^{+/+}$, *pole1*$^{+/m}$ and *pole1*$^{m/m}$ embryos were raised at the standard temperature of 28.5°C and subjected to whole-mount RNA in situ hybridization with *rag1*-specific and *gh*-specific probes and determination of the size of the retina (each symbol represents an individual fish) at 5 dpf. **b** Same as in (a), but fish were raised at 24°. **c** Comparison for the three parameters in (a) and (b) for the two temperatures; the values of wildtype fish were set to 1. **d** Ratio of parameter values illuminating the temperature sensitivity of T cell development in *pole1*$^{m/m}$ embryos. **e** Growth patterns of *S. cerevisiae* strains expressing wildtype *pol2* or mutant *pol2* (p.I648K). The plates were kept at 37°C for 48 hours and at 22°C for 72 hours, respectively. Scale bar, 1 cm. **f** Recovery of mouse embryos of the indicated genotypes at different time points; the day of the plug was counted as day E0.5 of gestation. The one homozygous mutant blastocyst was non-viable (marked by *).

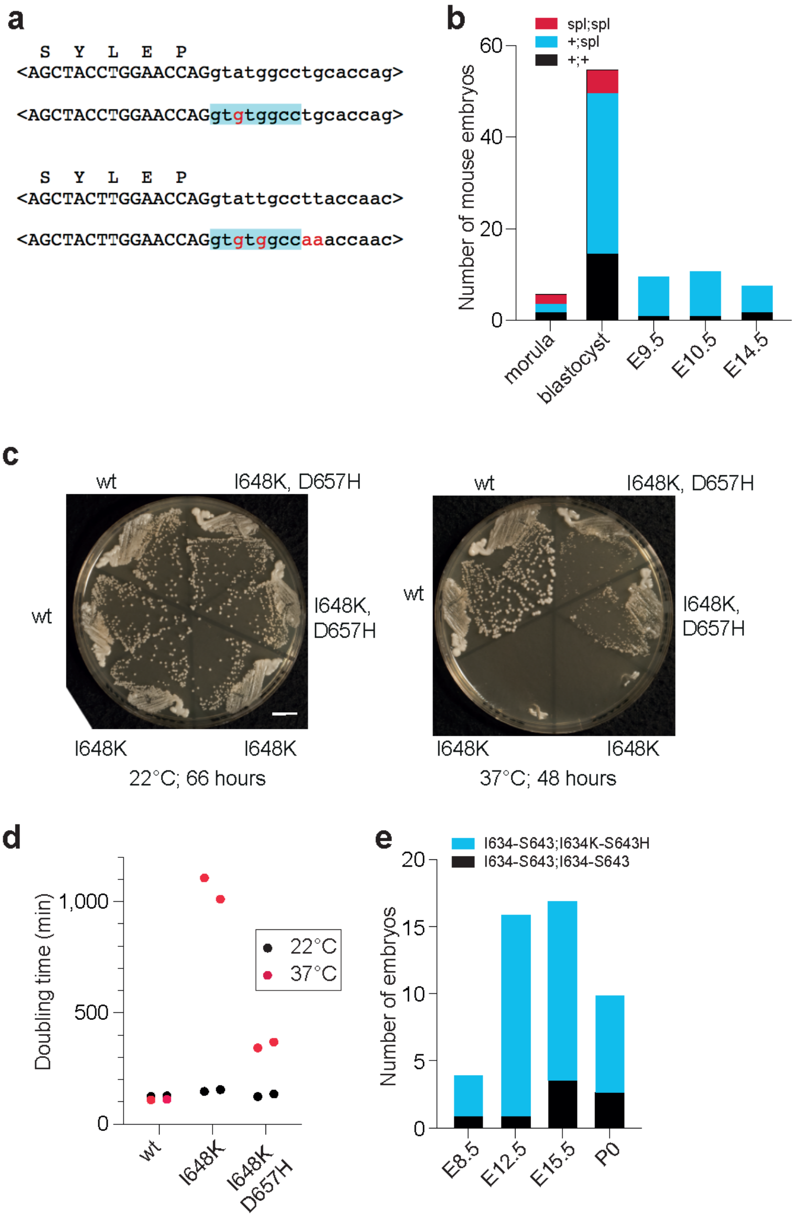

Supplementary Figure 2. Viability of additional *Pole1*/*Pol2* mutants. a Sequence context of *Pole1* exon 34 donor site corresponding to the site of a splice mutant observed in FILS syndrome patients; the 3′-ends of exon 34 are shown (upper case letters, including the conceptual translation in single letter code). The top two sequences are from the wildtype and mutant alleles of the affected human family, respectively; the A>G mutation in the intron (lower case letters) is indicated in red font. The bottom two sequences represent the mouse wild-type sequence and the CRISPR/Cas9-induced mutated sequence, respectively. **b** Recovery of mouse

embryos of the indicated genotypes at different time points; the day of the plug was counted as day E0.5 of gestation. **c** Growth pattern of wildtype *S. cerevisiae* strains, and strains carrying the indicated pol2 mutants; note that the double-mutant grows much faster than the p.I648K single mutant. Scale bar, 1cm. **d** Doubling times of *S. cerevisiae* strains carrying wildtype or mutant versions of pol2. **e** Recovery of mouse embryos of the indicated genotypes at different time points; the day of the plug was counted as day E0.5 of gestation. The I634K-S643H homozygous double mutant is not viable, indicating that the suppressor mutation identified in yeast pol2 has no beneficial effect on the I634K mutation in mouse embryogenesis; the S643H homozygous mutant exhibits normal viability.

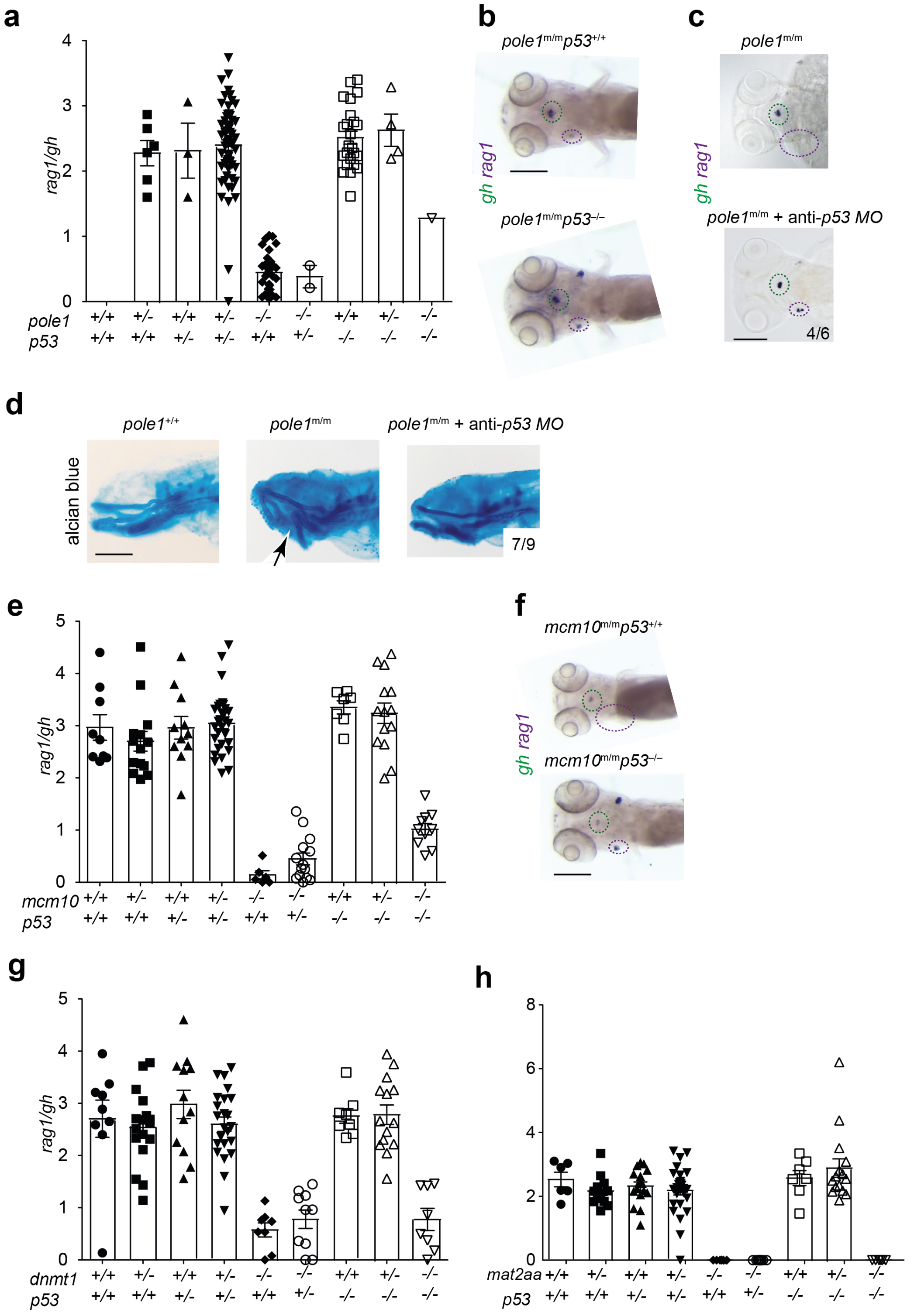

Supplementary Figure 3. The contribution of the p53 pathway to mutant phenotypes. a to **d** Genetic interaction analysis of the *pole1*^{t20320} allele. (a) Genetic interaction between *pole1* and *p53* at 5 dpf; the *rag1/gh* ratios of fish with the indicated genotypes are indicated (each symbol represents an individual fish). (b) Whole-mount RNA *in situ* hybridization results of larvae of the indicated genotypes are shown (*rag1* signal in thymus [indicated by purple circle]; *gh* signal in hypophysis [indicated by green circle]). (c) Activation of a p53-dependent check-point in *pole1*

mutants. Injection of an anti-*p53* oligonucleotide into *pole* homozygous mutants leads to the re-appearance of *rag1*-expressing cells in the thymus in 4 out 6 morphants, as determined at 4 dpf. (d) The malformed cartilaginous structures are normalized in 7 out of 9 morphants, as determined by alcian blue staining at 4 dpf (lateral view); a wild-type fish is shown for reference. **e** and **f** Genetic interaction between *mcm10* and *p53* at 5 dpf. (e) The *rag1/gh* ratios of fish with the indicated genotypes are indicated (each symbol represents an individual fish). (f) Whole-mount RNA *in situ* hybridization results of larvae of the indicated genotypes are shown (*rag1* signal in thymus [indicated by purple circle]; *gh* signal in hypophysis [indicated by green circle]). **g** Genetic interaction between *dnmt1* and *p53* at 5 dpf; the *rag1/gh* ratios of fish with the indicated genotypes are indicated (each symbol represents an individual fish). **h** Genetic interaction between *mat2aa* and *p53* at 5 dpf; the *rag1/gh* ratios of fish with the indicated genotypes are indicated (each symbol represents an individual fish). Scale bars (b, c, d, f), 1 mm.

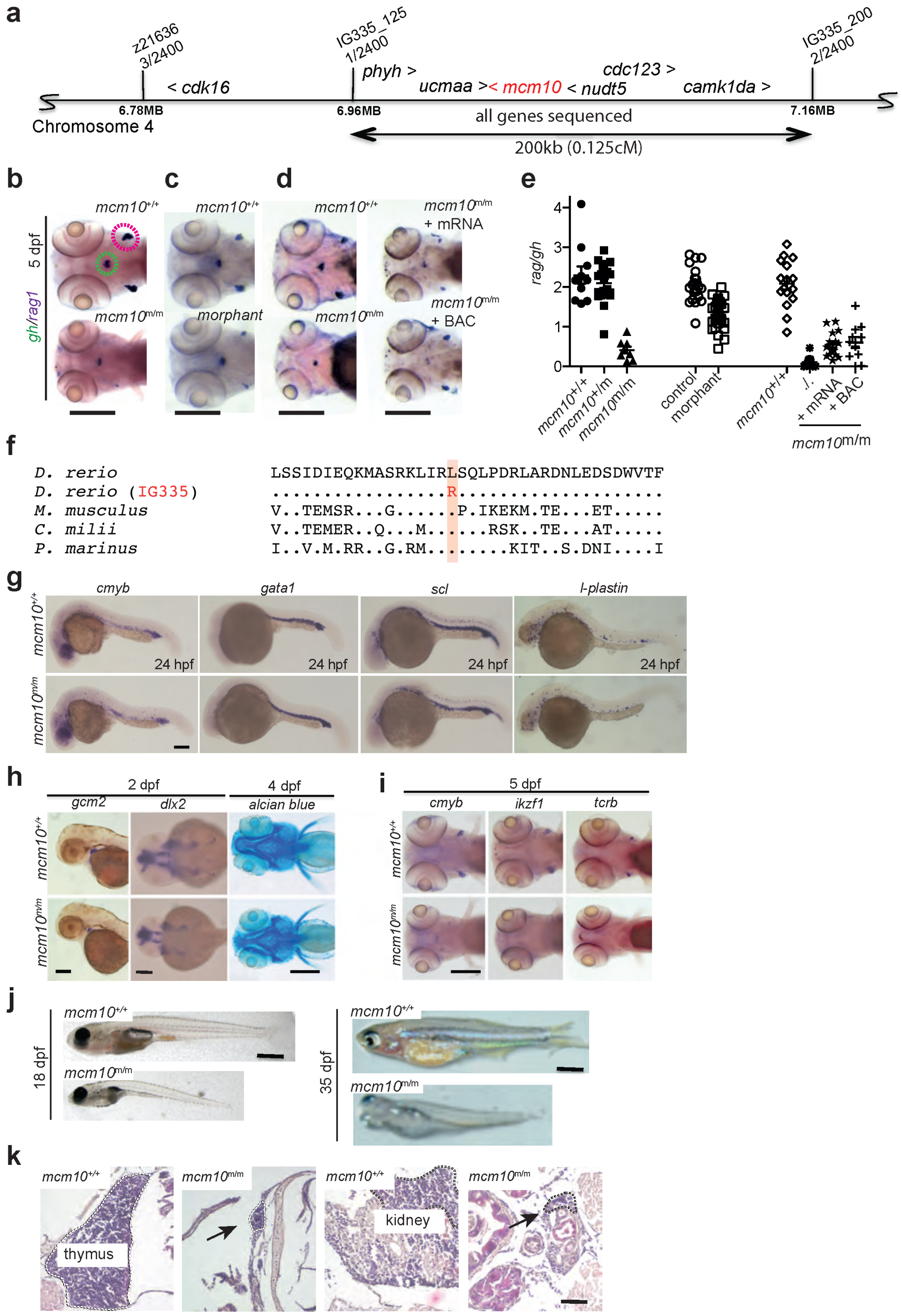

Supplementary Figure 4. Characterization of a zebrafish *mcm10* hypomorphic allele. a Genetic map for the region on chromosome 4 in the vicinity of the *mcm10* gene (not to scale). The number of recombination events observed between several genetic markers and the mutated locus in the number of meioses shown is indicated in brackets (for details on polymorphic markers, see Methods). The locations and transcriptional orientations of several genes relative to *mcm10* are also shown. **b** Diagnostic whole-mount RNA *in situ* hybridization using *rag1*, a marker for

differentiating thymocytes (thymus encircled in purple), and *gh*, detecting growth hormone gene expressing cells (green circle) in the hypophysis of zebrafish embryos at 5 days after fertilization (dpf); note the lack of *rag1* signals in mutant embryos, while *gh* signals are normal (dorsal views). **c** Phenocopy of the *mcm10* mutation in *mcm10* morphants. **d** Phenotypic rescue of *mcm10* mutation after injection of mouse *Mcm10* mRNA (top right) or a mouse BAC clone containing the *Mcm10* gene (bottom right). **e** Thymopoietic indices for fish with the indicated genotypes, including data for *mcm10* morphants, and the two types of rescue experiments (each symbol represents one fish). **f** Identification of an p.L248R missense mutation in the *mcm10* gene of IG335 mutants. Partial protein sequences of Mcm10 proteins of several vertebrate species are shown, with the universally conserved leucine residue indicated by shading. **g** Normal embryonic haematopoiesis in *mcm10* mutants, as determined by whole-mount RNA *in situ* hybridization at 24 hours after fertilization with the indicated gene-specific probes; representative of between 4 and 7 mutant embryos. **h** Craniofacial structures are mildly impaired as determined by alcian blue staining to visualize cartilage structures was performed at 4 dpf (representative of 3 mutants). Near normal craniofacial structures are also found as determined by whole-mount RNA *in situ* hybridization with *dlx2*, at 3 days after fertilization (dpf) (representative of 5 mutant embryos). Normal shape of pharyngeal ectoderm as visualized by g*cm2* expression; representative of 5 mutants. **i** Impaired T cell differentiation as determined by expression of *ikzf1, ccr9b* and *tcrb* (representative of between 5 and 15 mutants). **j** Growth retardation of *mcm10* mutants of the indicated ages; note the bloodless appearance of mutants. **k** Histological appearance of *mcm10* mutant tissues. Note the dwarfed thymus and the hypoplastic head kidney tissues indicative of impaired haematopoiesis. Scale bars (b, c, d, i, j [1mm]); (g, k [0.1mm]); h (gcm2, dlx2: 0.1mm; alcian blue, 0.8mm).

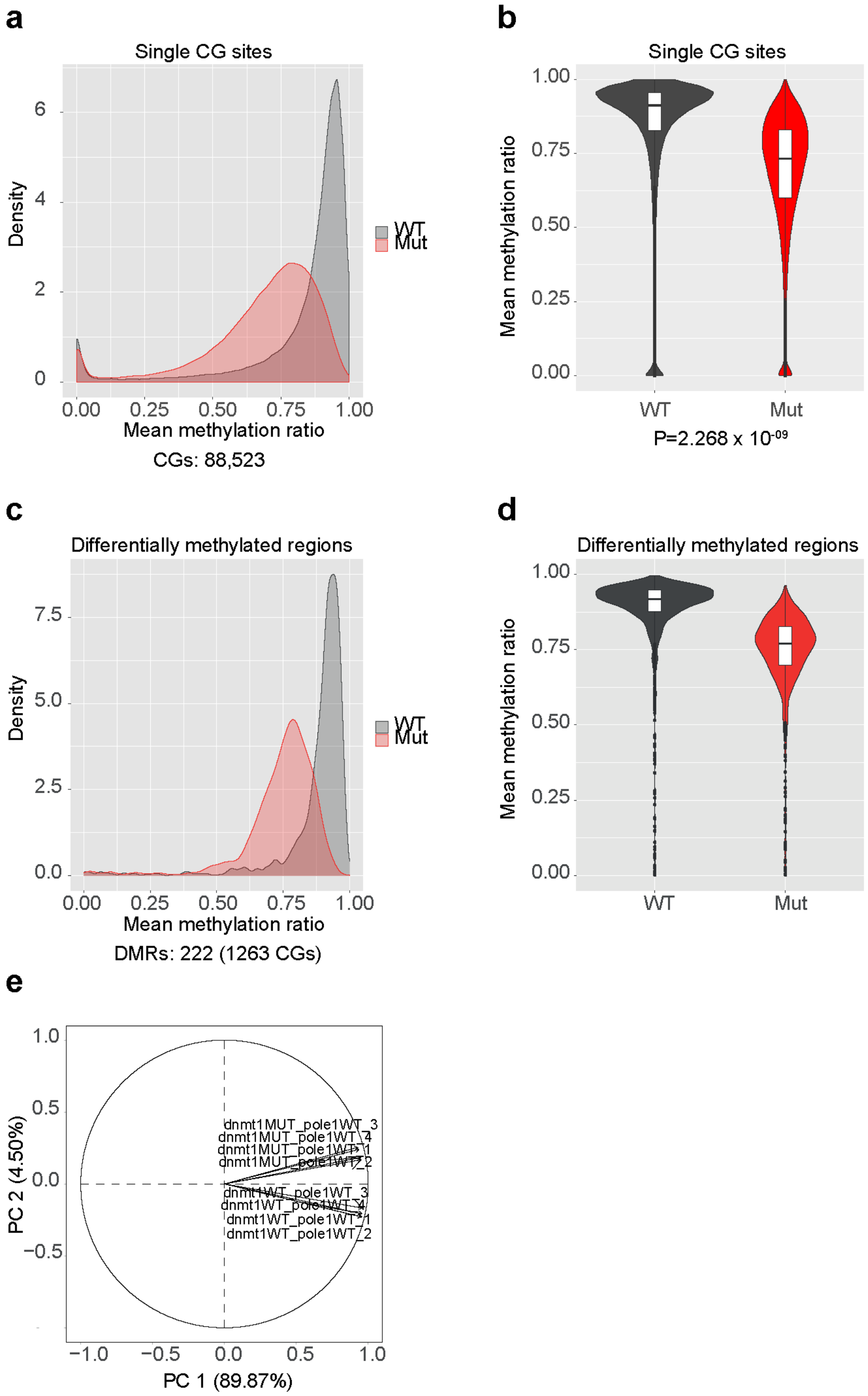

Supplementary Figure 5. Global reduction of CG DNA methylation levels in *dnmt1*$^{m/m}$ embryos. a Density plots of mean methylation ratios from wildtype (WT; *dnmt1*$^{+/+}$ *pole1*$^{+/+}$) and mutant (Mut; *dnmt1*$^{m/m}$ *pole1*$^{+/+}$); DNA was isolated at 5 dpf from whole zebrafish embryos; the number of CG sites that could be evaluated in both conditions is indicated. **b** Violin plots of methylation ratios shown in (a). The difference between mean methylation ratios is significant at P=2.268 x 10^{-9} (t-test, two-tailed). **c** Density plots of mean methylation ratios of differentially methylated regions; the number of DMRs sites that could be evaluated in both conditions is

indicated. **d** Violin plots of methylation ratios shown in (c); the number of single CGs included in the DMRs is indicated. **e** Principal component analysis of *dnmt1*$^{+/+}$ *pole1*$^{+/+}$ and *dnmt1*$^{m/m}$*pole1*$^{+/+}$ WGBS datasets (CGs), indicating the homogeneity of the four biological replicates.

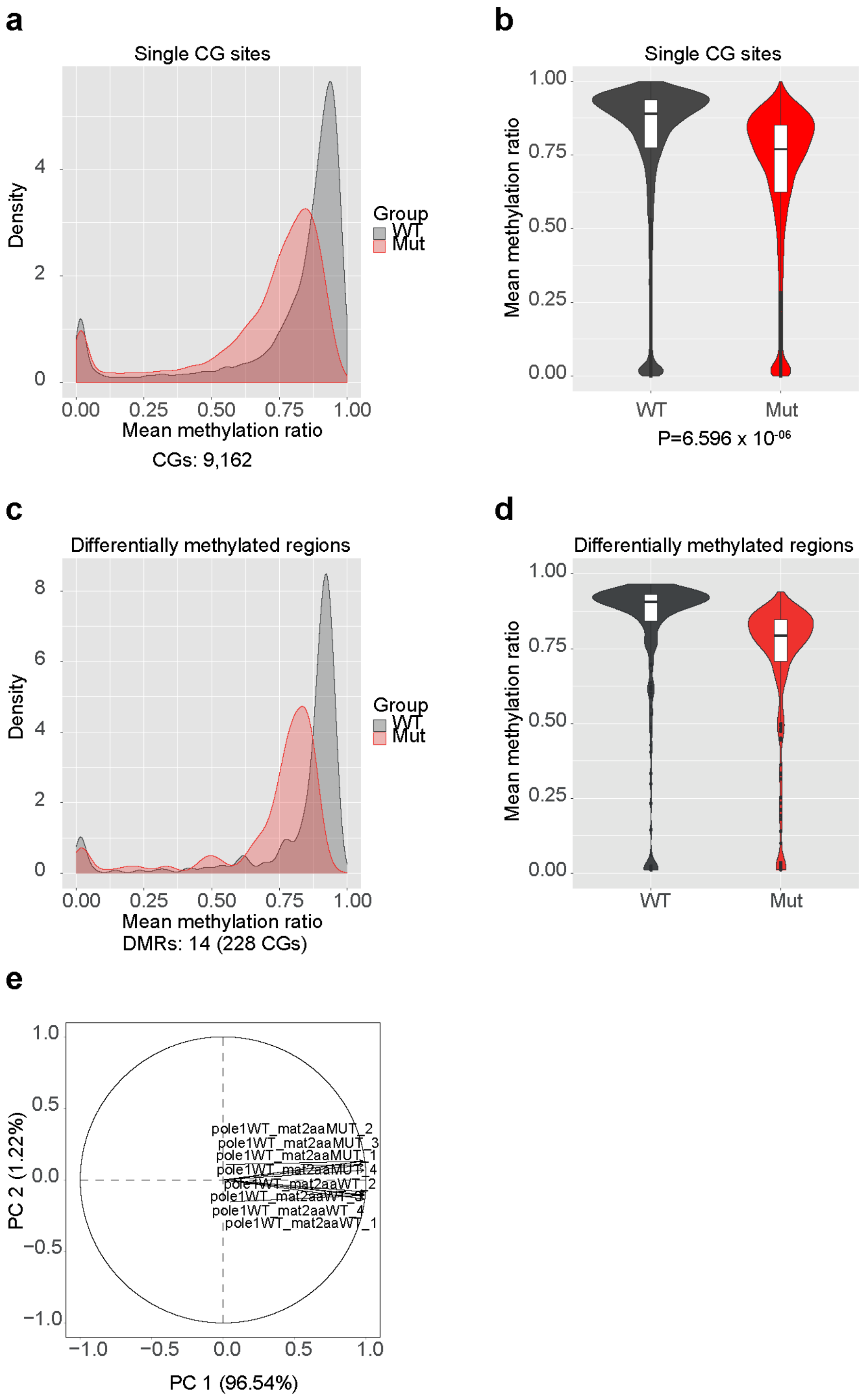

Supplementary Figure 6. Global decrease of CG DNA methylation levels in *mat2aa*$^{-/-}$ embryos. a Density plots of mean methylation ratios from wildtype (WT; *mat2aa*$^{+/+}$) and mutant (Mut; *mat2aa*$^{-/-}$); DNA was isolated at 5 dpf from whole zebrafish embryos; the number of CG sites that could be evaluated in both conditions is indicated. **b** Violin plots of methylation ratios shown in (a). The difference between mean methylation ratios is significant at P=6.596x10^{-6} (t-test, two-tailed). **c** Density plots of mean methylation ratios of differentially methylated regions; the number of DMRs sites that could be evaluated in both conditions is indicated. **d** Violin plots of

methylation ratios shown in (c); the number of single CGs included in the DMRs is indicated. **e** Principal component analysis of *mat2aa*$^{+/+}$and *mat2aa*$^{-/-}$ WGBS datasets (CGs), indicating the homogeneity of the four biological replicates.

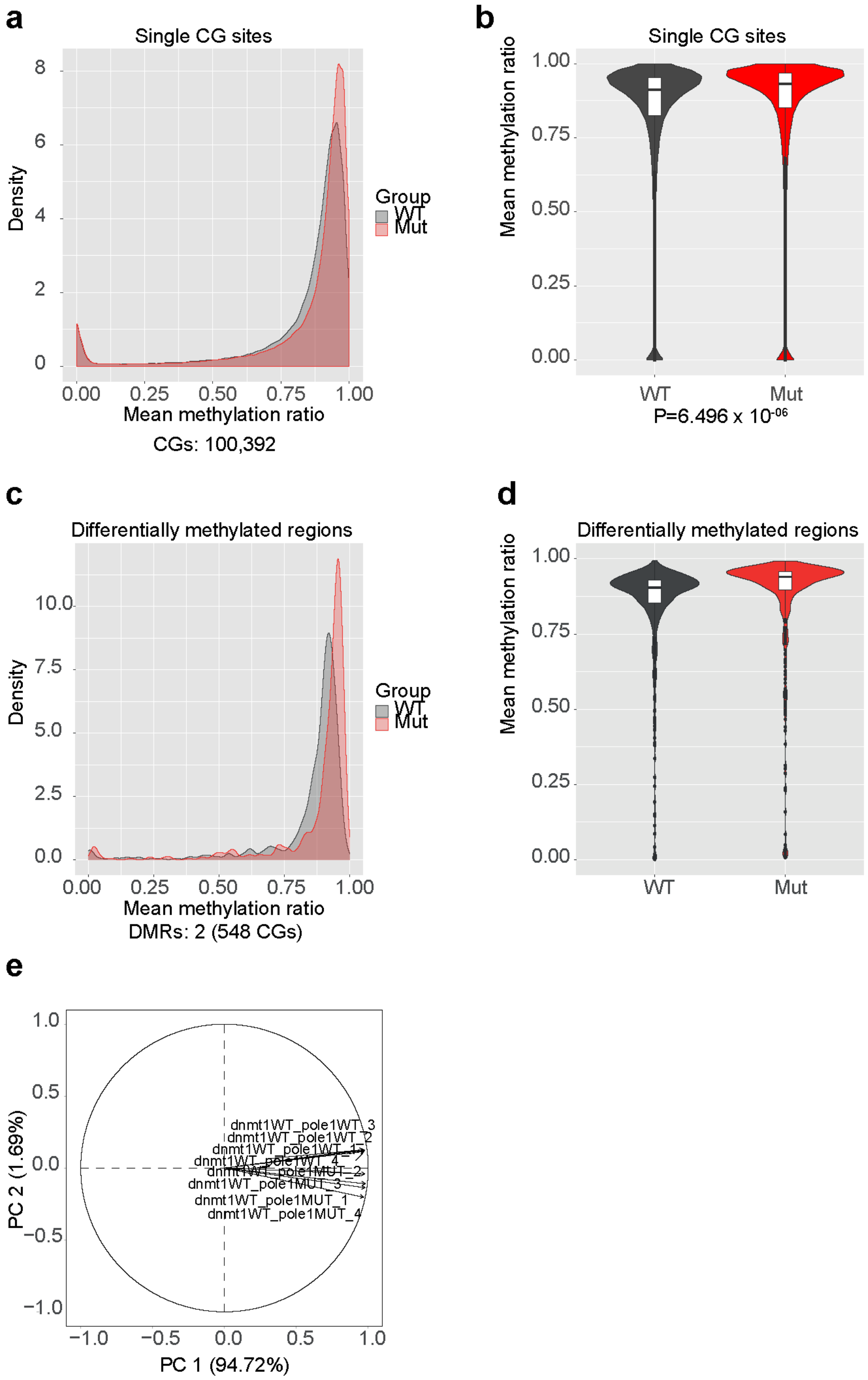

Supplementary Figure 7. Global increase of CG DNA methylation levels in *pole1*$^{m/m}$ embryos. a Density plots of mean methylation ratios from wildtype (WT; *dnmt1*$^{+/+}$ *pole1*$^{+/+}$) and mutant (Mut; *dnmt1*$^{+/+}$ *pole1*$^{m/m}$); DNA was isolated at 5 dpf from whole zebrafish embryos; the number of CG sites that could be evaluated in both conditions is indicated. **b** Violin plots of methylation ratios shown in (a). The difference between mean methylation ratios is significant at P=6.496 x 10^{-6} (t-test, two-tailed). **c** Density plots of mean methylation ratios of differentially methylated regions; the number of DMRs sites that could be evaluated in both conditions is

indicated. **d** Violin plots of methylation ratios shown in (c); the number of single CGs included in the DMRs is indicated. **e** Principal component analysis of *dnmt1*$^{+/+}$ *pole1*$^{+/+}$ and *dnmt1*$^{+/+}$ *pole1*$^{m/m}$ WGBS datasets (CGs), indicating the homogeneity of the four biological replicates.

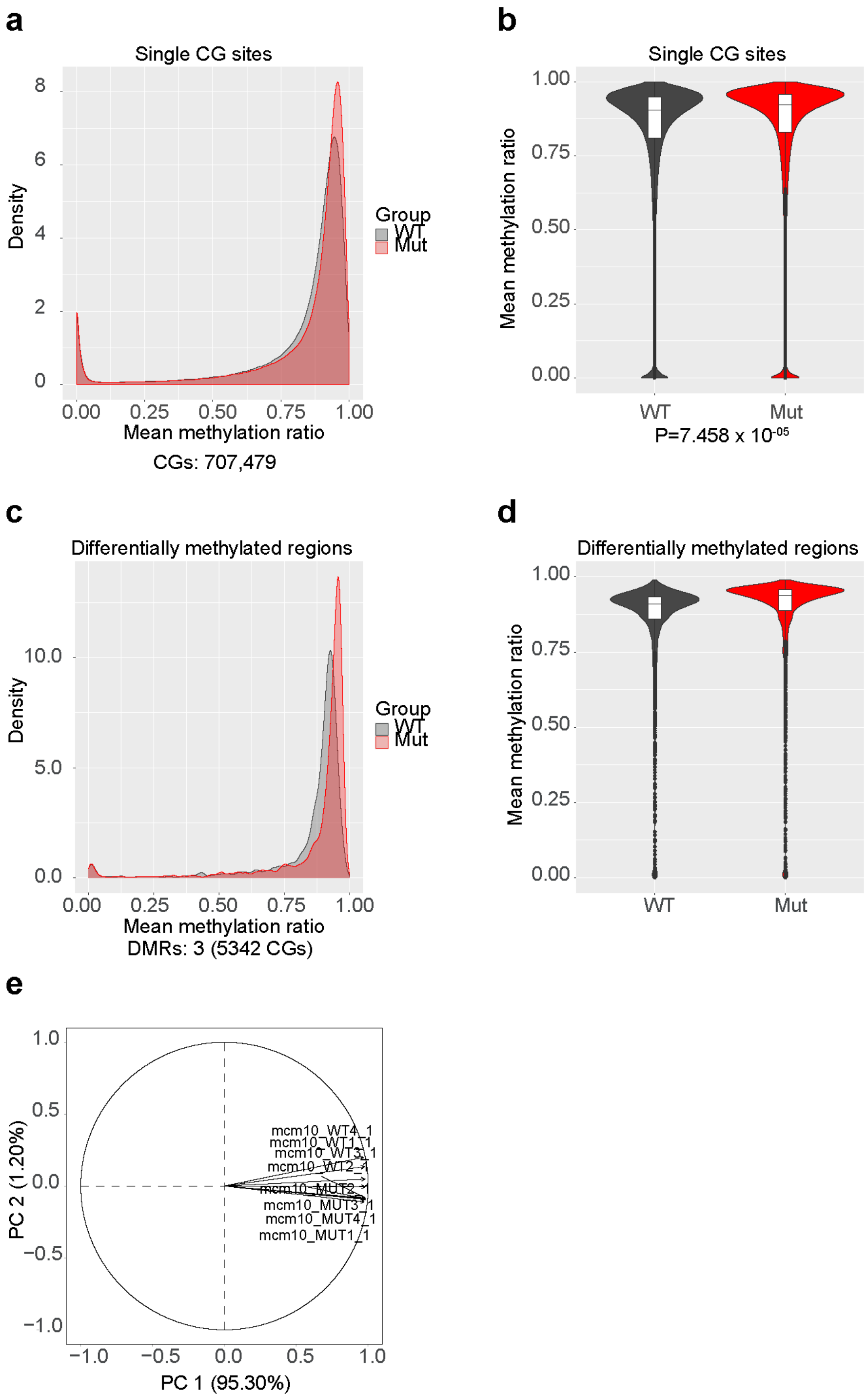

Supplementary Figure 8. Global increase of CG DNA methylation levels in *mcm10*$^{m/m}$ embryos. a Density plots of mean methylation ratios from wildtype (WT; *mcm10*$^{+/+}$) and mutant (Mut; *mcm10*$^{m/m}$); DNA was isolated at 5 dpf from whole zebrafish embryos; the number of CG sites that could be evaluated in both conditions is indicated. **b** Violin plots of methylation ratios shown in (a). The difference between mean methylation ratios is significant at P=7.458x 10^{-5} (t-test, two-tailed). **c** Density plots of mean methylation ratios of differentially methylated regions; the number of DMRs sites that could be evaluated in both conditions is indicated. **d** Violin plots of

methylation ratios shown in (c); the number of single CGs included in the DMRs is indicated. **e** Principal component analysis of *dnmt1*$^{+/+}$ *pole1*$^{+/+}$ and *dnmt1*$^{m/m}$ *pole1*$^{+/+}$ WGBS datasets (CGs), indicating the homogeneity of the four biological replicates.

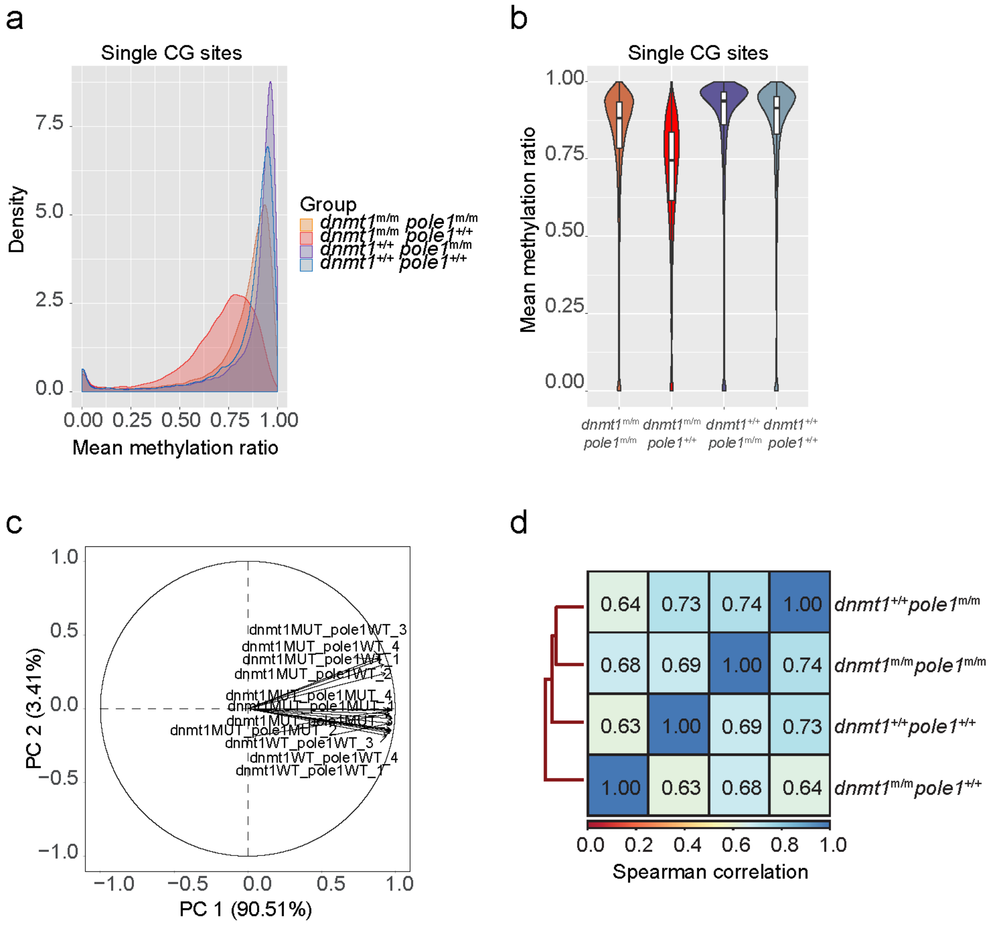

Supplementary Figure 9. Near-normal CG DNA methylation levels in *dnmt1*$^{m/m}$ *pole1*$^{m/m}$ double-mutant embryos. a Density plots of mean methylation ratios for the indicated genotypes; DNA was isolated at 5 dpf from whole zebrafish embryos. **b** Violin plots of methylation ratios shown in (a). **c** Principal component analysis of WGBS datasets (CGs), indicating the homogeneity of the four biological replicates. **d** Spearman correlation analysis of methylation ratios across the entire zebrafish genome.

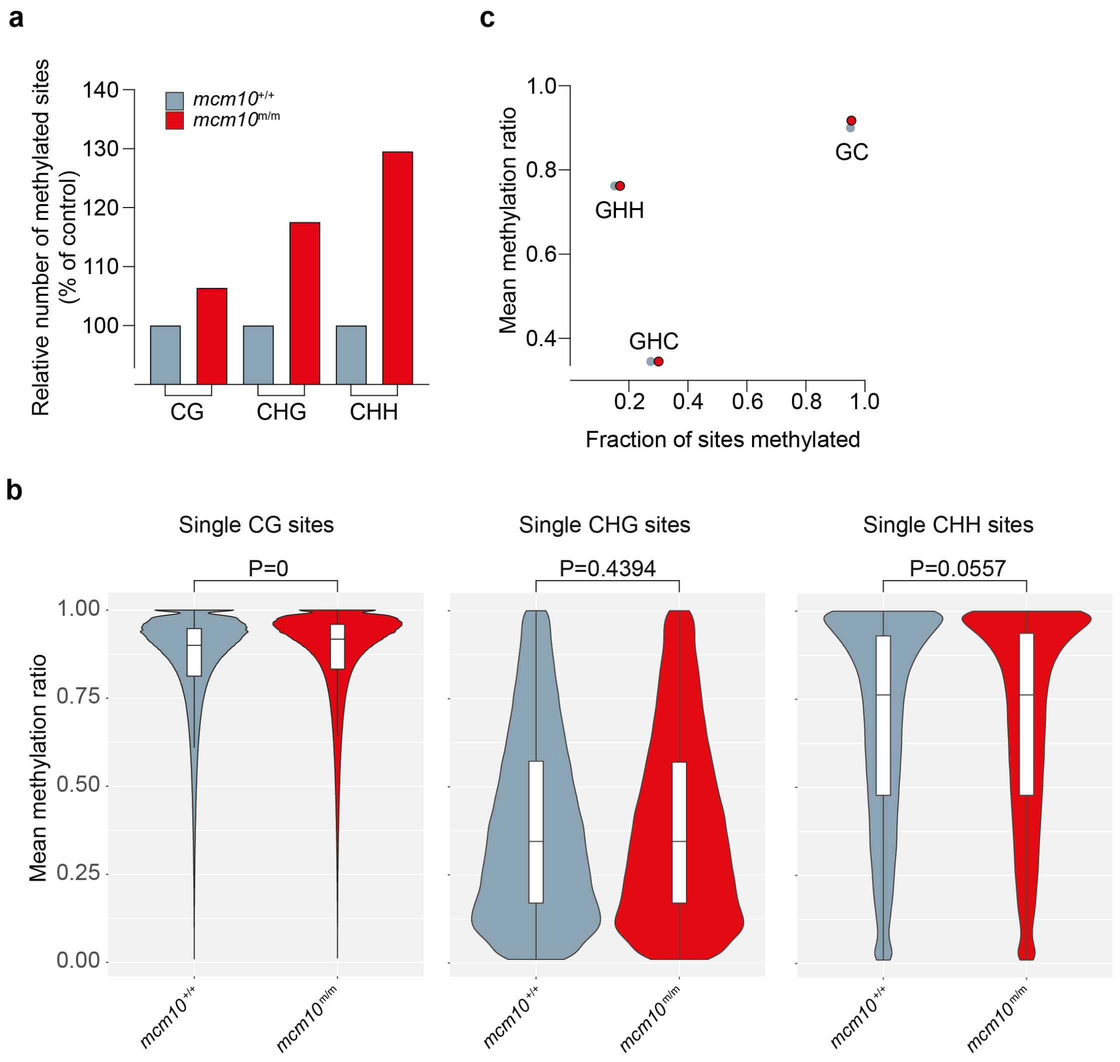

Supplementary Figure 10. Alterations in non-CG methylation patterns in *mcm10* mutants. a Increased numbers of methylated sites for CG dinucleotides, and CHG and CHH tri-nucleotides. The number of sites in wild-type fish is set to 100 (for absolute numbers, see Supplementary Table 1). **b** Violin plots of methylation ratios. The significance levels of the differences between mean methylation ratios are indicated (t-test, two-tailed). **c** Correlation plot between the fractions of sites methylated and their mean methylation ratios. For (b,c), colour codes of genotypes as in (a).

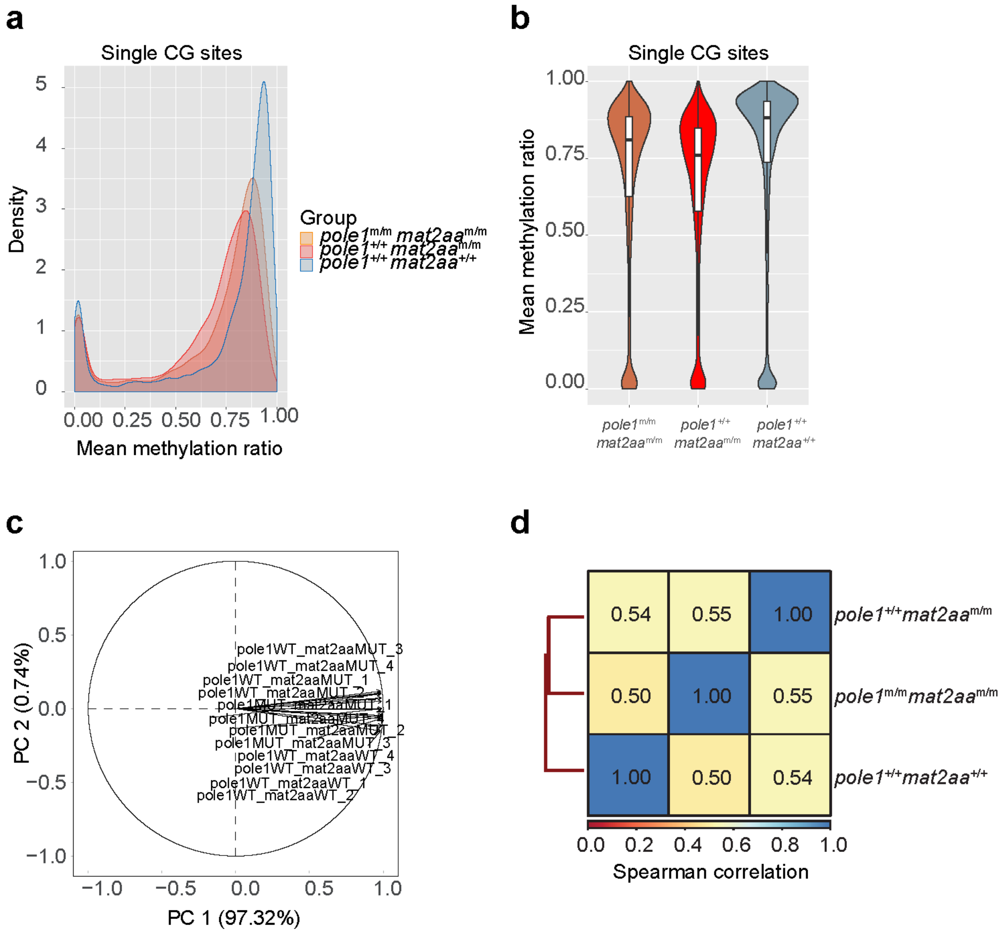

Supplementary Figure 11. Failure to normalize CG DNA methylation levels in *mat2aa*$^{-/-}$ *pole1*$^{m/m}$ double-mutant embryos. **a** Density plots of mean methylation ratios for the indicated genotypes; DNA was isolated at 5 dpf from whole zebrafish embryos. **b** Violin plots of methylation ratios shown in (a). **c** Principal component analysis of WGBS datasets (CGs), indicating the homogeneity of the four biological replicates. **d** Spearman correlation analysis of methylation ratios across the entire zebrafish genome.

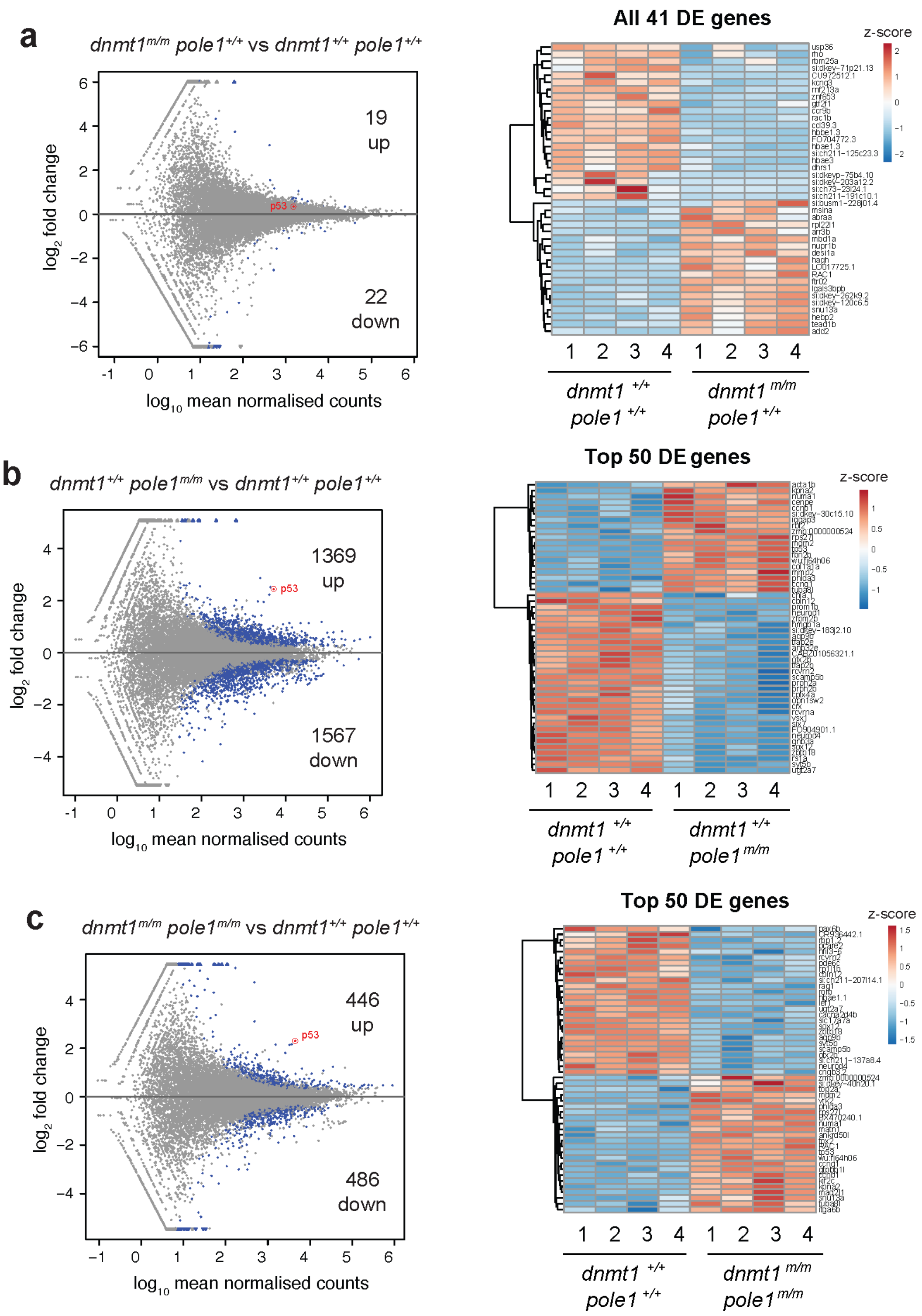
a
dnmt1m/m pole1+/+ vs dnmt1+/+ pole1+/+
19
up
22
down
p53
log2 fold change
log10 mean normalised counts
All 41 DE genes
z-score
dnmt1 +/+
pole1 +/+
dnmt1 m/m
pole1 +/+
b
dnmt1+/+ pole1m/m vs dnmt1+/+ pole1+/+
1369
up
1567
down
p53
log2 fold change
log10 mean normalised counts
Top 50 DE genes
z-score
dnmt1 +/+
pole1 +/+
dnmt1 +/+
pole1 m/m
c
dnmt1m/m pole1m/m vs dnmt1+/+ pole1+/+
446
up
486
down
p53
log2 fold change
log10 mean normalised counts
Top 50 DE genes
z-score
dnmt1 +/+
pole1 +/+
dnmt1 m/m
pole1 m/m

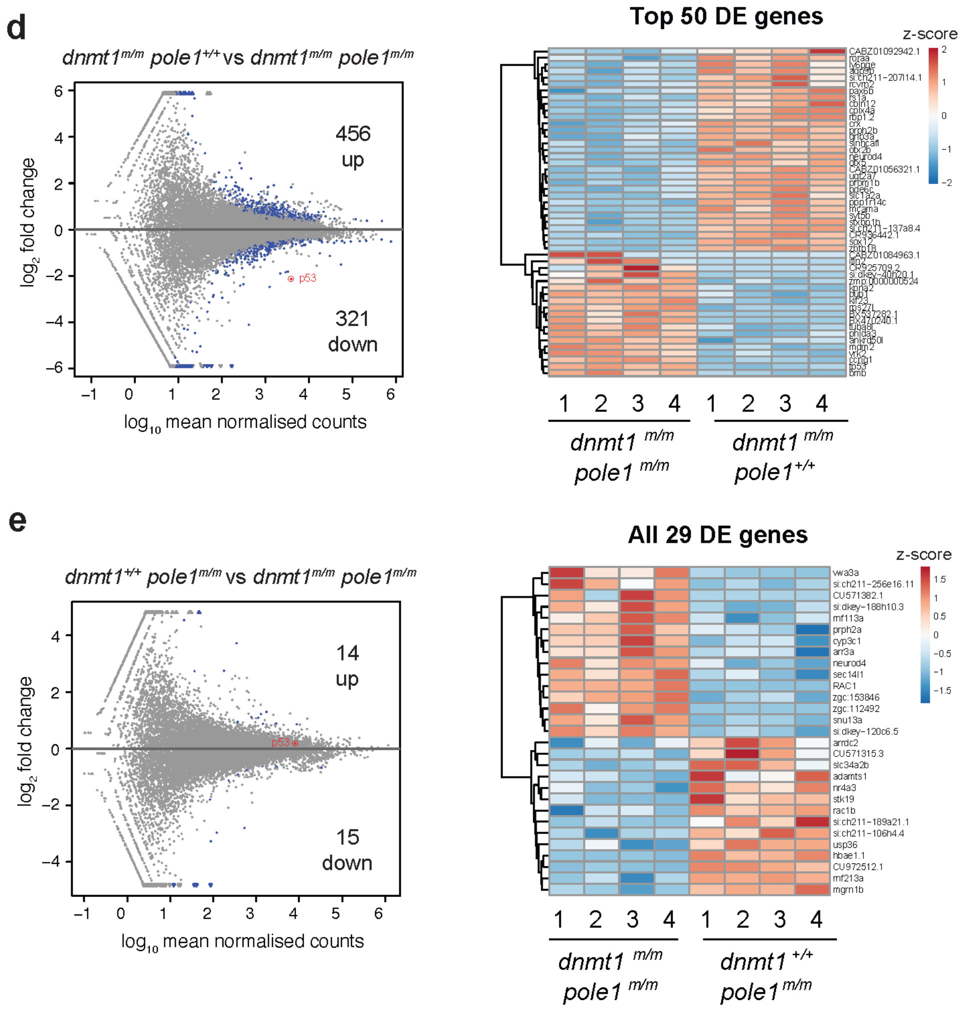

Supplementary Figure 12. Differential gene expression patterns in *dnmt1*$^{m/m}$*pole1*$^{m/m}$ double mutants. For each genotype, the number of up- and downregulated genes represented in an MA plot of mean values of normalized counts (x axis) versus $\log_2$ fold changes (y axis) and a heatmap representing k-means clustering (k=4) of row z-scores of $\log_2$ counts of differentially expressed (DE) genes across all datasets are shown. **a** Transcriptional changes in *dnmt1*$^{m/m}$ mutants against wildtype siblings; the heatmap shows all 41 differentially expressed genes. **b** Transcriptional changes in *pole1*$^{m/m}$ mutants against wildtype siblings; the heatmap shows the top 50 differentially expressed genes. **c** Transcriptional changes in *dnmt1*$^{m/m}$*pole1*$^{m/m}$ double mutants against wildtype siblings; the heatmap shows the top 50 differentially expressed genes. **d** Transcriptional changes in *dnmt1*$^{m/m}$*pole1*$^{m/m}$ double mutants against *dnmt1*$^{m/m}$ single mutant siblings; the heatmap shows the top 50 differentially expressed genes. **e** Transcriptional changes in *dnmt1*$^{m/m}$*pole1*$^{m/m}$ double mutants against *pole1*$^{m/m}$ single mutant siblings; the heatmap shows all 29 differentially expressed genes, indicating that the transcriptional changes elicited by the *pole1*$^{m/m}$ condition dominate those in *dnmt1*$^{m/m}$*pole1*$^{m/m}$ double mutants.

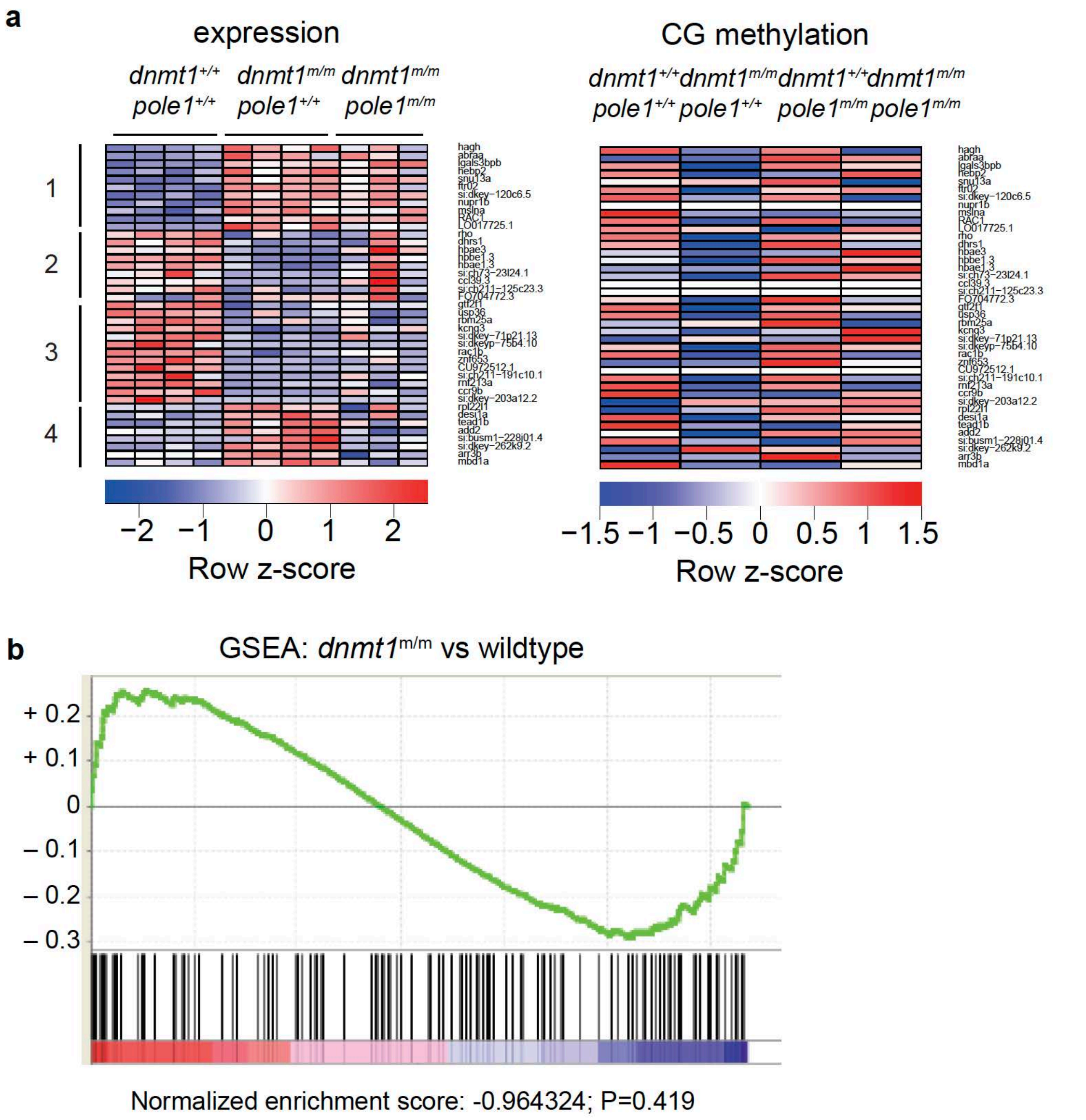

Supplementary Figure 13. Lack of correlation between expression levels and CG methylation levels for genes differentially expressed in *dnmt1*$^{m/m}$ single mutants and *dnmt1*$^{m/m}$*pole* $^{m/m}$ double mutants. a Restoration of aberrant expression levels in 2 groups of genes in double mutants (see also Fig. 7); methylation levels in gene bodies of differentially expressed genes. **b** Enrichment plot indicating the lack of correlation between expression and methylation levels calculated using the GSEA algorithm.

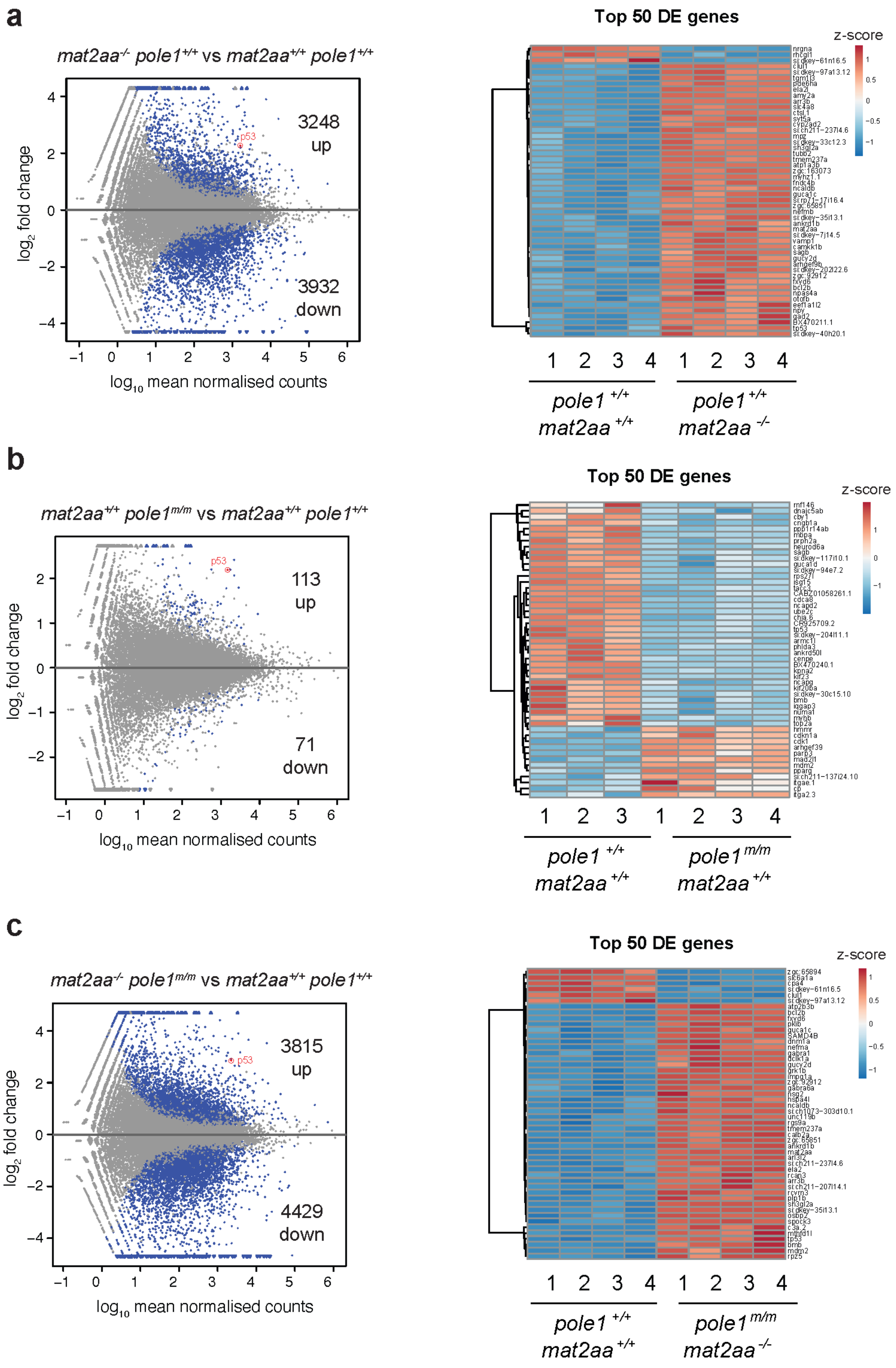

a
mat2aa-/- pole1+/+ vs mat2aa+/+ pole1+/+
3248 up
3932 down
p53
log2 fold change
log10 mean normalised counts
Top 50 DE genes
z-score
pole1 +/+ mat2aa +/+
pole1 +/+ mat2aa -/-
b
mat2aa+/+ pole1m/m vs mat2aa+/+ pole1+/+
113 up
71 down
p53
log2 fold change
log10 mean normalised counts
Top 50 DE genes
z-score
pole1 +/+ mat2aa +/+
pole1 m/m mat2aa +/+
c
mat2aa-/- pole1m/m vs mat2aa+/+ pole1+/+
3815 up
4429 down
p53
log2 fold change
log10 mean normalised counts
Top 50 DE genes
z-score
pole1 +/+ mat2aa +/+
pole1 m/m mat2aa -/-

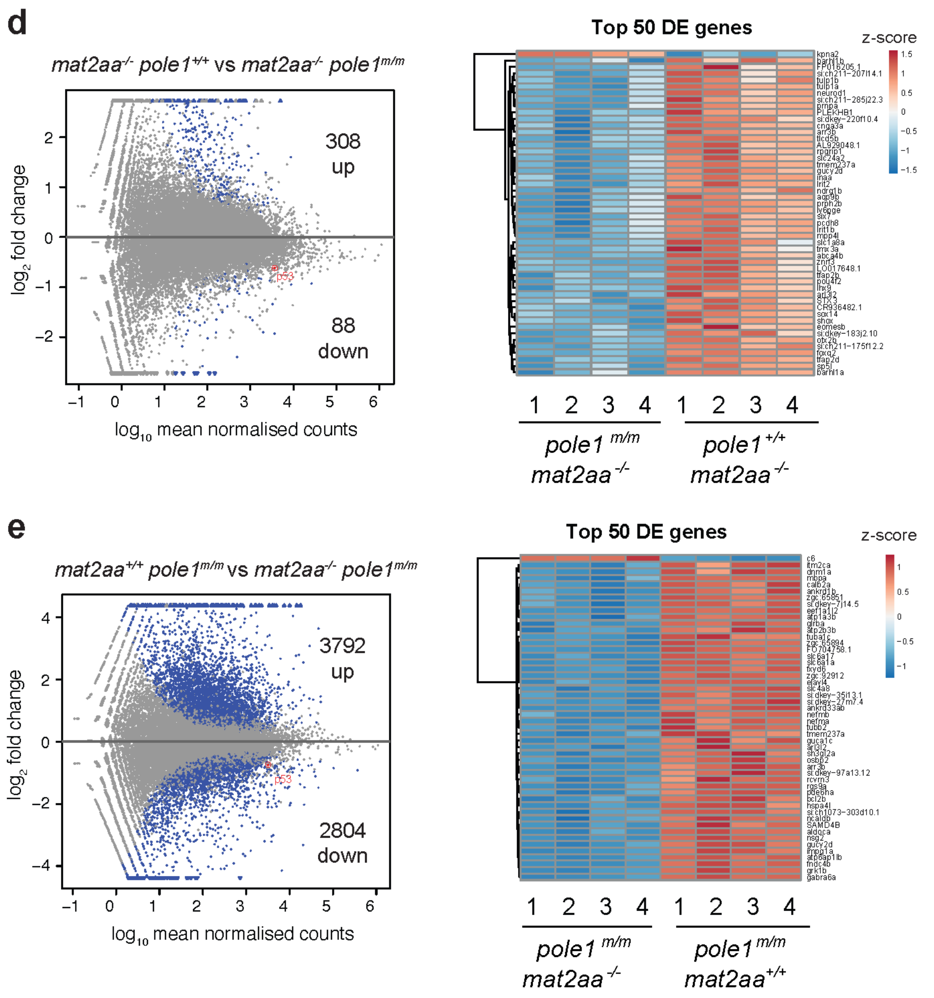

Supplementary Figure 14. Differential gene expression patterns in *mat2aa*$^{-/-}$ *pole1*$^{m/m}$ double-mutants. For each genotype, the number of up- and downregulated genes represented in an MA plot of mean of normalized counts (x axis) versus $\log_2$ fold change (y axis) and a heatmap representing k-means clustering (k=4) of row z-scores of $\log_2$ counts of differentially expressed (DE) genes across all datasets are shown. **a** Transcriptional changes in *mat2aa*$^{-/-}$ mutants against wildtype siblings; the heatmap shows the top 50 differentially expressed genes. **b** Transcriptional changes in *pole1*$^{m/m}$ mutants against wildtype siblings; the heatmap shows the top 50 differentially expressed genes. **c** Transcriptional changes in *mat2aa*$^{-/-}$*pole1*$^{m/m}$ double mutants against wildtype siblings; the heatmap shows the top 50 differentially expressed genes. **d** Transcriptional changes in *mat2aa*$^{-/-}$*pole1*$^{m/m}$ double mutants against *mat2aa*$^{-/-}$ single mutant siblings; the heatmap shows the top 50 differentially expressed genes. **e** Transcriptional changes in *mat2aa*$^{-/-}$*pole1*$^{m/m}$ double mutants against *pole1*$^{m/m}$ single mutant siblings; the heatmap shows the top 50 differentially expressed genes.

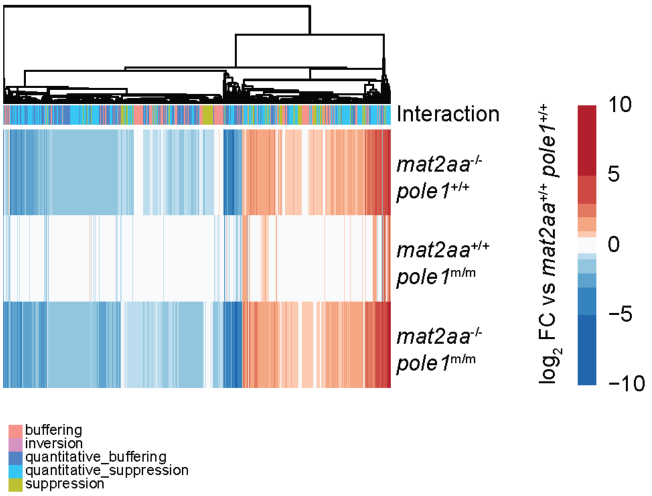

Supplementary Figure 15. Expression levels of differentially expressed genes in *mat2aa* mutants are not restored in *mat2aa*$^{-/-}$*pole1*$^{m/m}$ double mutants. Global gene expression analysis in the indicated genotypes, demonstrating that expression changes in *mat2aa* and *pole1* mutants do not overlap and that the *mat2aa* mutant transcriptome is not altered in double mutants. The type of genetic interaction follows the nomenclature in[86].

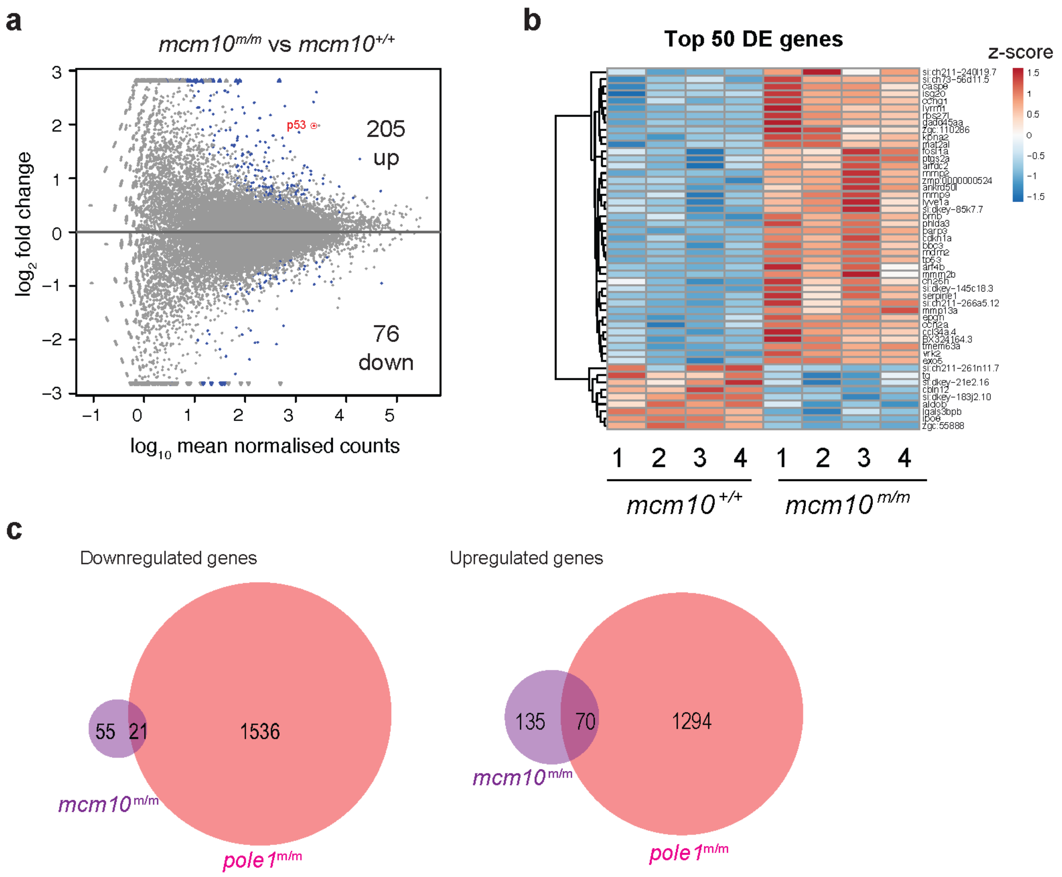

Supplementary Figure 16. Differential gene expression patterns in *mcm10* and *pole1* mutants. a MA plot of transcriptional changes in *mcm10*$^{m/m}$ mutants against wildtype siblings **b** Heatmap showing the top 50 differentially expressed (DE) genes; the heatmap represents k-means clustering (k=4) of row z-scores of $\log_2$ counts of differentially expressed genes across all datasets. **c** Venn diagram comparing down-regulated and up-regulated genes in *mcm10*$^{m/m}$ and *pole1*$^{m/m}$ mutants.

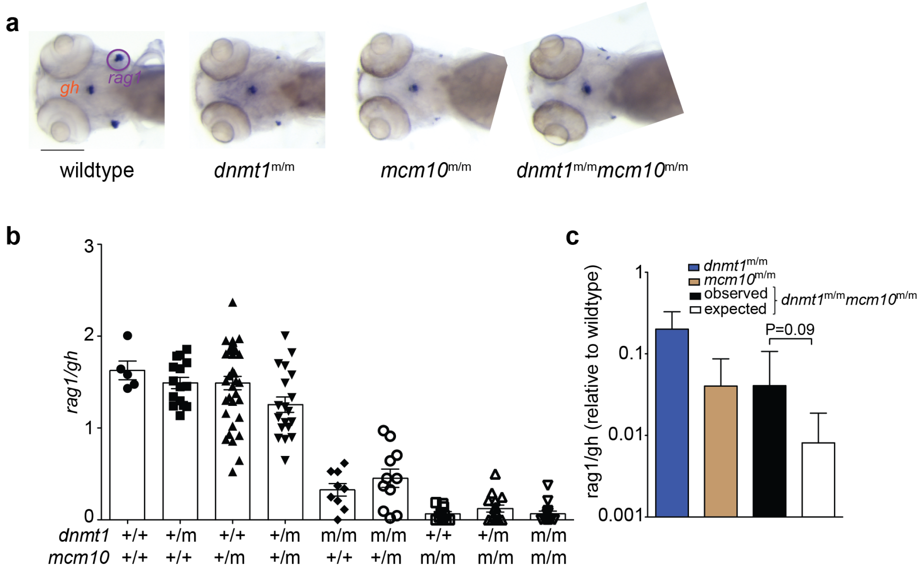

Supplementary Figure 17. Genetic interaction between *dnmt1* and *mcm10*. a Whole-mount RNA *in situ* hybridization results of larvae of the indicated genotypes are shown (*rag1* signal in thymus [indicated by purple circle]; *gh* signal in hypophysis). The lens and retina are outlined. Scale bar, 1mm. **b** Representation of *rag1/gh* ratios of fish with the indicated genotypes (each symbol represents an individual fish) (mean±sem). **c** Quantitative analysis of data in (b) (mean±sem; t-test; two-tailed); modest alleviating interaction is demonstrated. Values in (b) are normalized to wild-type; expected values were calculated by multiplying the values of the two single mutants with error propagation, and compared against observed values.

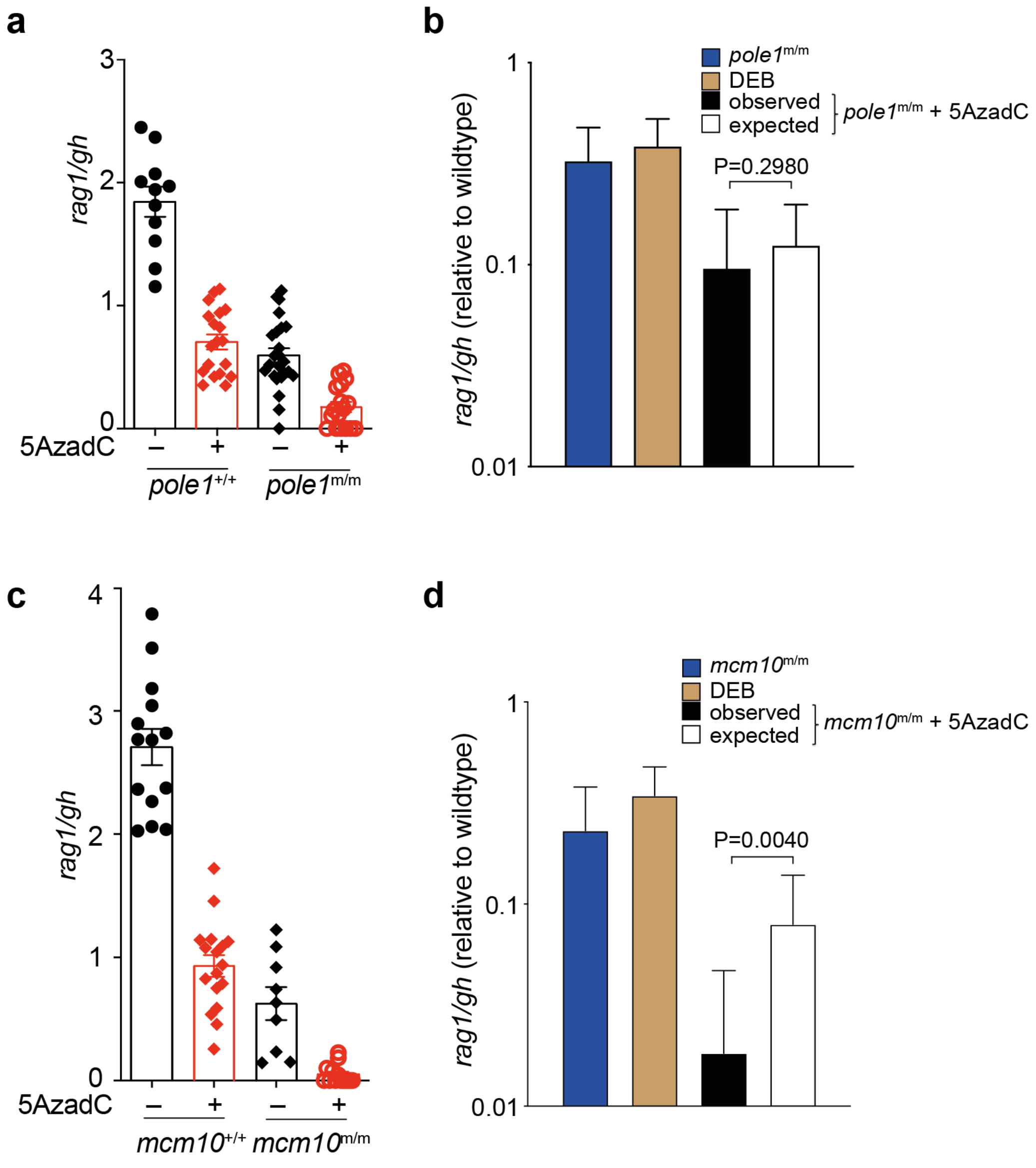

Supplementary Figure 18. Genetic interaction between *pole1*, *mcm10* and the DNA methylation inhibitor 5´-aza-2'-deoxycytidine (5AzadC). a Representation of *rag1/gh* ratios of fish with the indicated *pole1* genotypes exposed to solvent or 5 µM 5AzadC (each symbol represents an individual fish). **b** Quantitative analysis of data in (a) (mean±sem; t-test; two-tailed); no significant interaction is demonstrated. Values in (a) are normalized to wild-type; expected values were calculated by multiplying the values of the two single conditions with error propagation, and compared against observed values. **c** Representation of *rag1/gh* ratios of fish with the indicated *mcm10* genotypes exposed to solvent or 5 µM 5AzadC (each symbol represents an individual fish). **d** Quantitative analysis of data in (c) (mean±sem; t-test; two-tailed); synthetic interaction is demonstrated. Values in (b) are normalized to wild-type; expected values were calculated by multiplying the values of the two single conditions with error propagation, and compared against observed values.

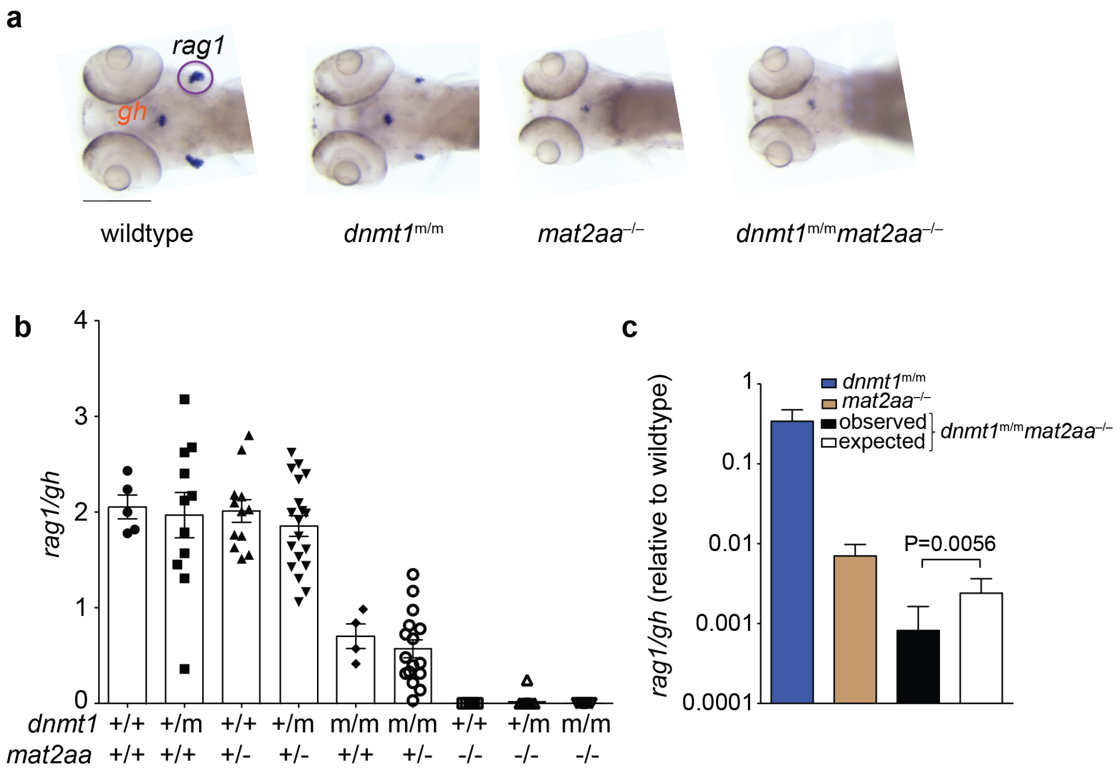

Supplementary Figure 19. Genetic interaction between *dnmt1* and *mat2aa*. a Whole-mount RNA *in situ* hybridization results of larvae of the indicated genotypes are shown (*rag1* signal in thymus [indicated by purple circle]; *gh* signal in hypophysis). The lens and retina are outlined. Scale bar, 1mm. **b**, Representation of *rag1/gh* ratios of fish with the indicated genotypes (each symbol represents an individual fish) (mean±sem). **c** Quantitative analysis of data in (b) (mean±sem; t-test; two-tailed); synthetic interaction is demonstrated. Values in (b) are normalized to wild-type; expected values were calculated by multiplying the values of the two single mutants with error propagation, and compared against observed values.

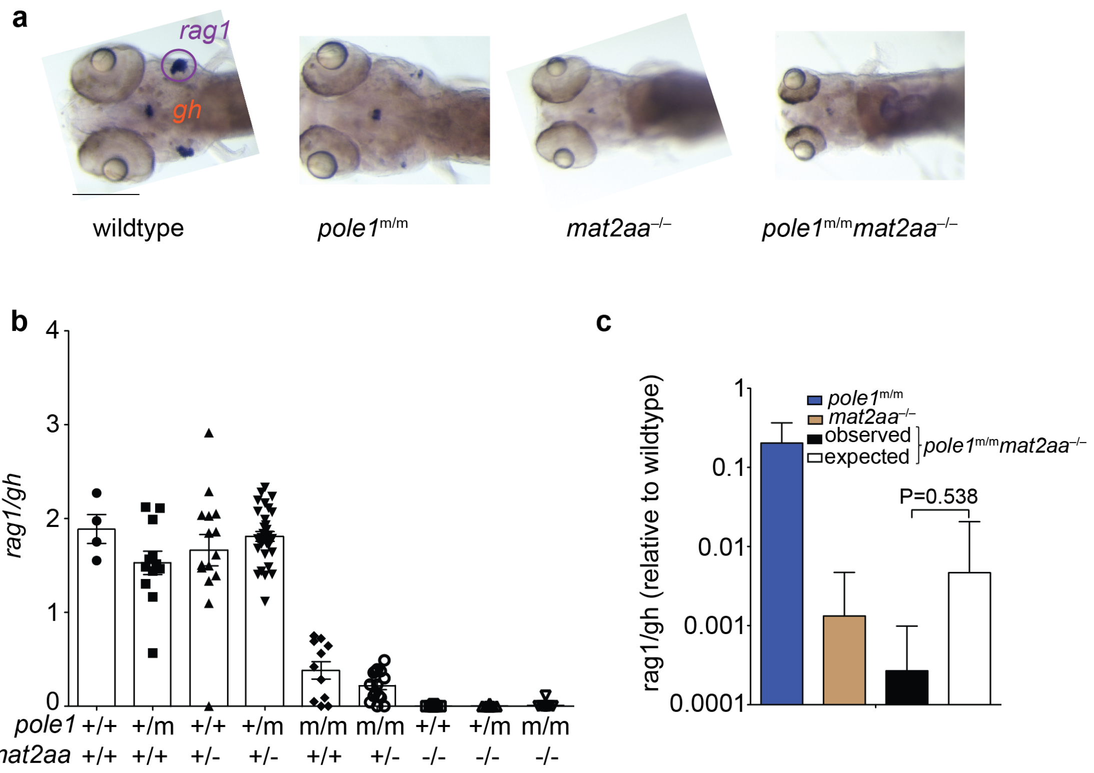

Supplementary Figure 20. Genetic interaction between *mat2aa* and *pole1*. a Whole-mount RNA *in situ* hybridization results of larvae of the indicated genotypes are shown (*rag1* signal in thymus [indicated by purple circle]; *gh* signal in hypophysis). The lens and retina are outlined. Scale bar, 1mm. **b** Representation of *rag1/gh* ratios of fish with the indicated genotypes (each symbol represents an individual fish) (mean±sem). **c** Quantitative analysis of data in (b) (mean±sem; t-test; two-tailed); synthetic interaction is demonstrated. Values in (b) are normalized to wild-type; expected values were calculated by multiplying the values of the two single mutants with error propagation, and compared against observed values.

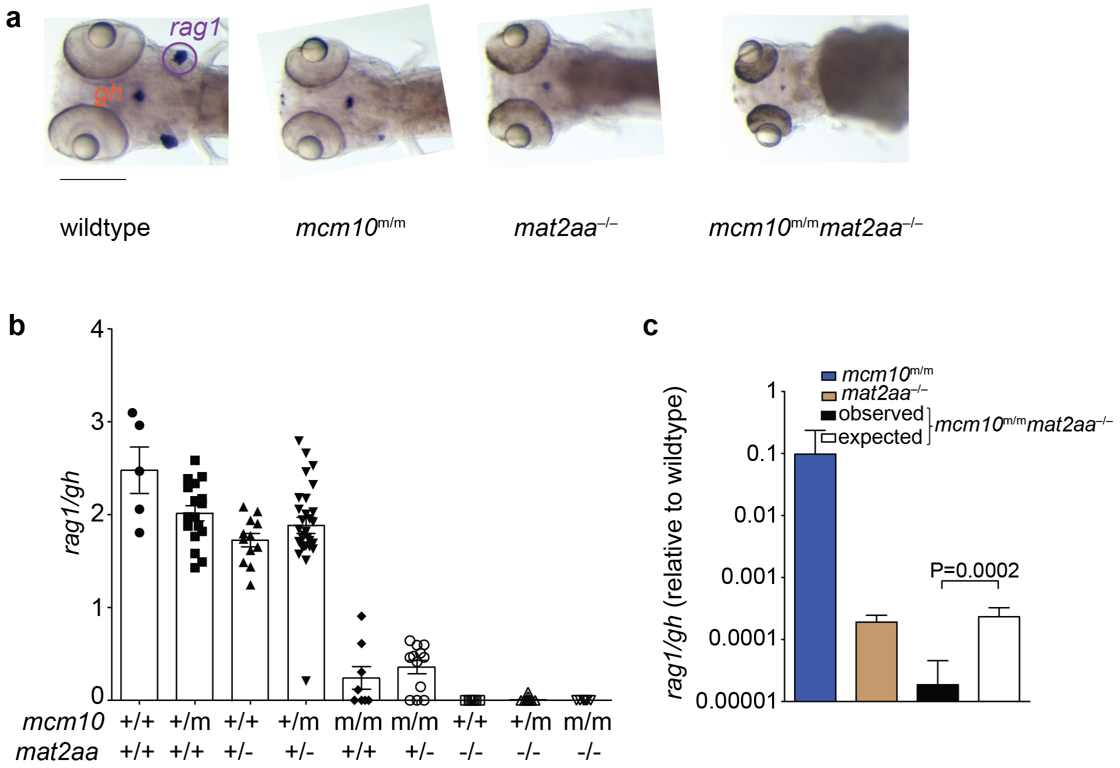

Supplementary Figure 21. Genetic interaction between *mat2aa* and *mcm10*. a Whole-mount RNA *in situ* hybridization results of larvae of the indicated genotypes are shown (*rag1* signal in thymus [indicated by purple circle]; *gh* signal in hypophysis). The lens and retina are outlined. Scale bar, 1mm. **b** Representation of *rag1/gh* ratios of fish with the indicated genotypes (each symbol represents an individual fish) (mean±sem). **c** Quantitative analysis of data in (b) (mean±sem; t-test; two-tailed); synthetic interaction is demonstrated. Values in (b) are normalized to wild-type; expected values were calculated by multiplying the values of the two single mutants with error propagation, and compared against observed values.

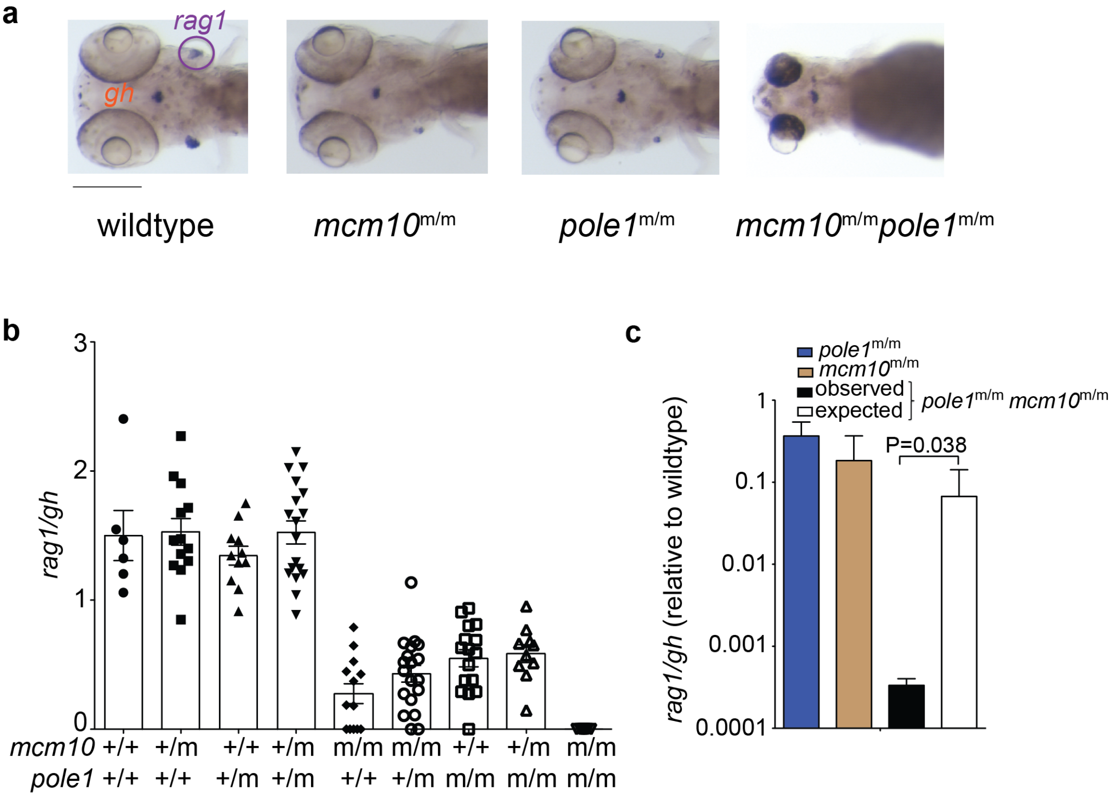

Supplementary Figure 22. Genetic interaction between *mcm10* and *pole1*. a Whole-mount RNA *in situ* hybridization results of larvae of the indicated genotypes are shown (*rag1* signal in thymus [indicated by purple circle]; *gh* signal in hypophysis). The lens and retina are outlined. Scale bar, 1mm. **b** Representation of *rag1/gh* ratios of fish with the indicated genotypes (each symbol represents an individual fish) (mean±sem). **c** Quantitative analysis of data in (b) (mean±sem; t-test; two-tailed); synthetic interaction is demonstrated. Values in (b) are normalized to wild-type; expected values were calculated by multiplying the values of the two single mutants with error propagation, and compared against observed values.

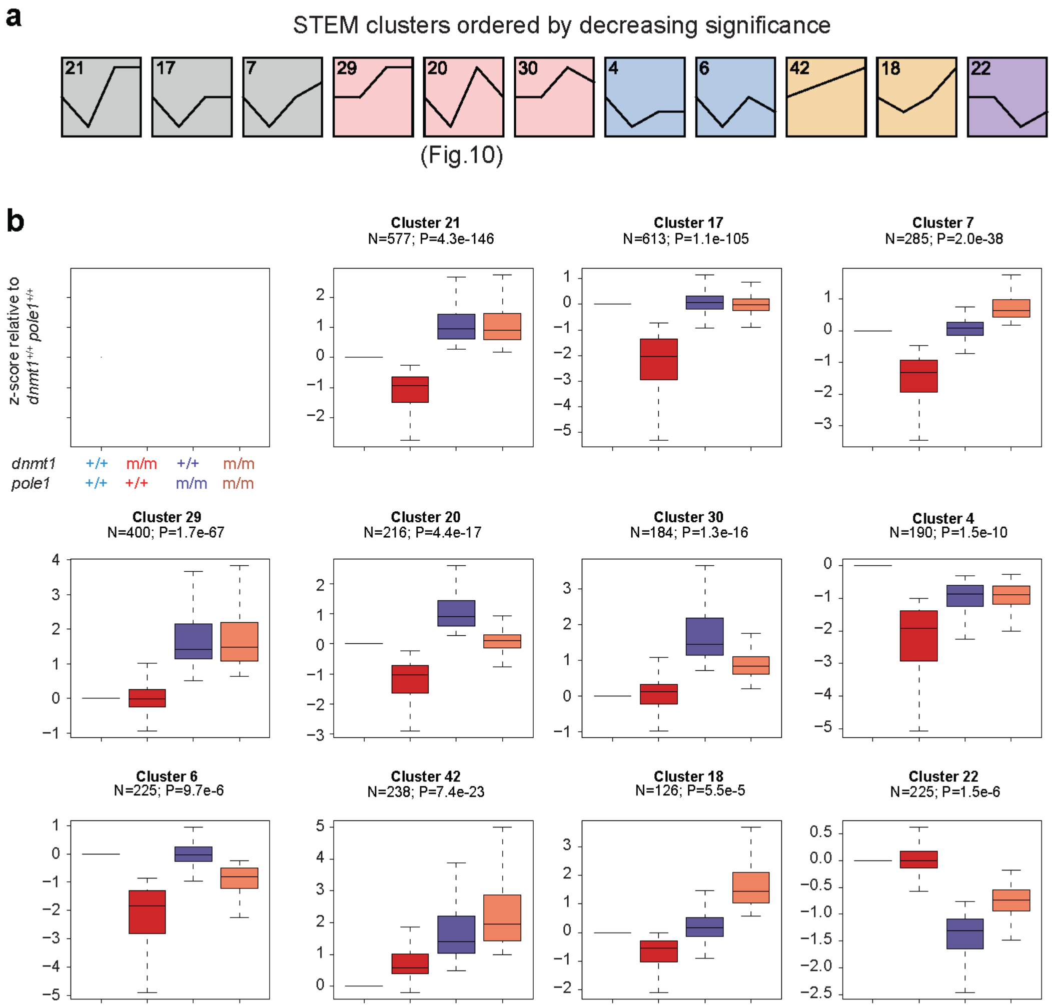

Supplementary Figure 23. STEM (Short Time-series Expression Miner) analysis of co-regulated genes across dnmt1 and pole1 single and double mutants. a Schematic display of gene clusters showing significant trends ordered by decreasing P-value. Cluster 20 is presented in more detail in Fig. 10. **b** Detailed representation of patterns shown in (a); the numbers of genes per cluster are indicated. Box plots (mean; box, upper/lower quartile; whiskers, minimal/maximal values). A schematic indicating the order of genotypes in the other graphs is shown. The y axis displays the scores of mutants relative to the wildtype genotype.

Supplementary Table 1. Methylation analysis

	CG sites		CHG sites	CHH sites		
	total	methylated	total	methylated	total	methylated
**Group 1**						
dnmt1+/+pole1+/+	14916016	13981508	31282843	6018997	86089612	9951872
dnmt1m/mpole1+/+	14704010	13675347	30623623	5007134	84422892	8401596
dnmt1+/+pole1m/m	14867597	13974191	31138040	6796755	85904272	11522898
dnmt1m/mpole1m/m	14772711	13814516	30775043	5522611	83947066	9007201
**Group 2**						
mat2aa+/+pole1+/+	20320029	19761972	46858954	19025353	206341608	45617428
mat2aa-/-pole1+/+	19953385	19243515	45572003	16242422	189294300	36821332
mat2aa-/-pole1+/+	19517433	18743296	44225152	14602622	171187214	30305133
**Group 3**						
mcm10+/+	17531076	16679376	37986447	10438202	126202077	19172151
mcm10m/m	18592529	17752032	40768000	12275904	145485287	24842659
