# Supplementary material for: Antagonistic interactions safeguard mitotic propagation of genetic and epigenetic information in zebrafish
Source: Commun Biol. 2024 Jan 5;7:31. doi: 10.1038/s42003-023-05692-3 (PMC10770094; doi:10.1038/s42003-023-05692-3)
Supplement: Supplementary file 4 — Reporting Summary [file 42003_2023_5692_MOESM4_ESM.pdf]

nature portfolio

Corresponding author(s): Thomas Boehm

Last updated by author(s): Dec 1, 2023

Reporting Summary

Nature Portfolio wishes to improve the reproducibility of the work that we publish. This form provides structure for consistency and transparency in reporting. For further information on Nature Portfolio policies, see our Editorial Policies and the Editorial Policy Checklist.

Statistics

For all statistical analyses, confirm that the following items are present in the figure legend, table legend, main text, or Methods section.

n/a	Confirmed	
☐	☒	The exact sample size (*n*) for each experimental group/condition, given as a discrete number and unit of measurement
☐	☒	A statement on whether measurements were taken from distinct samples or whether the same sample was measured repeatedly
☐	☒	The statistical test(s) used AND whether they are one- or two-sided<br>*Only common tests should be described solely by name; describe more complex techniques in the Methods section.*
☒	☐	A description of all covariates tested
☒	☐	A description of any assumptions or corrections, such as tests of normality and adjustment for multiple comparisons
☐	☒	A full description of the statistical parameters including central tendency (e.g. means) or other basic estimates (e.g. regression coefficient) AND variation (e.g. standard deviation) or associated estimates of uncertainty (e.g. confidence intervals)
☒	☐	For null hypothesis testing, the test statistic (e.g. *F*, *t*, *r*) with confidence intervals, effect sizes, degrees of freedom and *P* value noted<br>*Give P values as exact values whenever suitable.*
☒	☐	For Bayesian analysis, information on the choice of priors and Markov chain Monte Carlo settings
☒	☐	For hierarchical and complex designs, identification of the appropriate level for tests and full reporting of outcomes
☒	☐	Estimates of effect sizes (e.g. Cohen's *d*, Pearson's *r*), indicating how they were calculated

Our web collection on statistics for biologists contains articles on many of the points above.

Software and code

Policy information about availability of computer code

Data collection

Nucleotide libraries were sequenced on an Illumina HiSeq 2500 according to manufacturer's instructions.

Data analysis

RNA-Seq processing. RNA-Seq processing was performed using snakePipes 2.4.2 (Ref. 70). Reads were aligned to the GRCz11 genome using STAR version 2.4.7a (Ref. 71) using --outStd BAM_Unsorted --outSAMtype BAM Unsorted --outSAMunmapped Within--sjdbGTFfile --sjdbOverhang 100 as parameters. Mapped reads were weeded for PCR duplicates using sambamba version 1.0.0 (Ref. 72) using --sort-buffer-size=6000 --overflow-list -size 600000 as a parameter.Counts were obtained using featureCounts from the subread package version 2.0.0 (Ref. 73) using -p -B -C -Q 10 --primary -T 8 -s 2 -a as parameters. Read coverages were obtained using deeptools bamCoverage version 3.3.2 (Ref. 74), with --binSize 25 --normalizeRPKM --effectiveGenomeSize 1679186873 --maxFragmentLength 1000 --scaleFactor as parameters following deeptools multiBamSummary version 3.3.2 using bins --scalingFactors as parameters. Differential gene expression analysis: differentially expressed genes were obtained DESEq2 version 1.26.0 (Ref. 75) using alpha = 0.05 as a parameter. Final normalised, r-log transformed count values were obtained using the rlog function. For clustering analyses, row z-scores of rlog transformed data for dnmt1+/+/pole1+/+; dnmt1m/m/pole1+/+ and dnmt1m/m/pole1m/m datasets for all 41 dnmt1m/m/pole1+/+; dnmt1m/m;pole1+/+ differentially regulated genes. k-means clustering was performed using the kmeans R function76. The Short Time-series Expression Miner (STEM) algorithm was used for the clustering of co-regulated genes56.
Gene ontology analysis. The analyses were performed using Homer findgo.pl (Ref. 77) with -bg zebrafish.base.gene as a parameter.

WGBS processing. WGBS processing was performed using snakePipes 1.2.3 (Ref. 70). Reads were adapter-, quality- and end- trimmed using cutadapt 2.1 (Ref. 78) using -a AGATCGGAAGAGC -A AGATCGGAAGAGC -q 10 -m 30 -j 8 as parameters. Reads were aligned to the bisulfite-converted GRCz11 genome using bwa-meth version 0.2.2 (Ref. 79) using --read-group as a parameter. Mapped reads were weeded for PCR duplicates using sambamba version 0.6.6 using --remove-duplicates as a parameter. CpG methylation extraction and coverages were obtained

using methyl_extract version 1.9 (Ref. 80) using default parameters. Differential methylation analysis was performed using metilene 0.2.6 (Ref. 81) with default parameters. Average coverage values were computed using the bedtools merge function (Ref. 82) with -c 4 -o mean as parameters on bedGraph files obtained from the bigWigToBedGraph function (Ref. 83). Spearman correlation clustering was performed using deeptools multiBigWigSummary bins function followed by plotCorrelation using --corMethod spearman and --removeOutliers as parameters. Average methylation profiles were generated using deeptools computeMatrix using --referencePoint TSS -b 3000 -a 3000 as parameters, followed by plotProfile.
Differentially-methylated regions (DMRs) were computed via metilene using the following parameters: maxDist: 300 minCpGs: 10 minCoverage: 5 FDR: 0.1 minMethDiff: 0.1. Note that our analysis cannot distinguish between hemimethylated and fully methylated cytosine residues.
For pair-wise comparisons, methylation coverages and ratios were computed from CpG positions in the reference genome with a minimum coverage specified by --minCoverage and low SNP allelic frequency (<0.25 illegitimate bases) in both conditions.

Comparison of CpG, CHG and CHH methylation levels. MethylDackel CpG, CHG and CHH methylation coverage files were obtained via snakePipes 2.4.2 using WGBS --DAG -j 10 -i <FASTQ> -o <output_folder> GRCz11.yaml --trim --MethylDackelOptions "--CHH --CHG --mergeContext --maxVariantFrac 0.25 --minDepth 4". To reduce skewing in violin plots due to high numbers of 0 methylation sites in CHG and CHH coverages, CpG, CHG and CHH coverage files were then filtered against 0, covered values. In the case of the mat2aa/pole1 CHH triplet, stringency was increased to values less than 25% methylation to account for very high numbers of fixed value artefacts between 0 and 25%. CpG, CHG and CHH coverage files were then sequentially processed in snakePipes 1.2.3 as methXT files and in the case of CHG and CHH files, renamed as “CpG” to produce violin plots for each condition group using WGBS --DAG -j 10 -i <FASTQ> -o <output_folder> GRCz11.yaml --sampleSheet <samplesheet>. The number of CpG, CHG and CHH sites numbers was derived by obtaining the union of all sites for all replicates per condition via bedtools multiinter -i <all_replicate_coverage_files_per_condition> | wc -l. Median and m.a.d. values were derived in R using apply(metilene.IN, 2, “<mean | mad>” from intermediary “merged_methylation_data_XXX/metilene.IN.txt” files representing the intersection of coverages for all replicates in all conditions per group, i.e. that shown in violin plots.

Gene set enrichment analysis. Gene set enrichment analyses were performed using GSEA version 4.0.3 (Ref. 84) with gene_set permutation type, weighted enrichment statistic, Diff_of_classes as the ranking metric on log2 transformed gene expression data.

Gene set enrichment analysis
Gene set enrichment analyses were performed using GSEA version 4.0.3 (Ref. 76) with gene_set permutation type, weighted enrichment statistic, Diff_of_classes as the ranking metric on log2 transformed gene expression data.

For manuscripts utilizing custom algorithms or software that are central to the research but not yet described in published literature, software must be made available to editors and reviewers. We strongly encourage code deposition in a community repository (e.g. GitHub). See the Nature Portfolio guidelines for submitting code & software for further information.

Data

Policy information about availability of data

All manuscripts must include a data availability statement. This statement should provide the following information, where applicable:

- Accession codes, unique identifiers, or web links for publicly available datasets
- A description of any restrictions on data availability
- For clinical datasets or third party data, please ensure that the statement adheres to our policy

RNA-Seq and WGBS data have been deposited at the Gene Expression Omnibus (GEO) under accessions GSE181571 and GSE181572, respectively. WGS bisulfite sequence analysis files deposited under 10.6084/m9.figshare.24711120 (Figshare)

Research involving human participants, their data, or biological material

Policy information about studies with human participants or human data. See also policy information about sex, gender (identity/presentation), and sexual orientation and race, ethnicity and racism.

Reporting on sex and gender	n/a
Reporting on race, ethnicity, or other socially relevant groupings	n/a
Population characteristics	n/a
Recruitment	n/a
Ethics oversight	n/a

Note that full information on the approval of the study protocol must also be provided in the manuscript.

Field-specific reporting

Please select the one below that is the best fit for your research. If you are not sure, read the appropriate sections before making your selection.

☒ Life sciences ☐ Behavioural & social sciences ☐ Ecological, evolutionary & environmental sciences

For a reference copy of the document with all sections, see nature.com/documents/nr-reporting-summary-flat.pdf

Life sciences study design

All studies must disclose on these points even when the disclosure is negative.

Sample size	Sample size was determined based on previous results with the animal system used.
Data exclusions	No animals were excluded from analyses.
Replication	Biological replicas (N≥3) were used; statistical tsts were done on biological replicas, not on technical replicas
Randomization	No randomization of animals was done in the present studies.
Blinding	Phenotypes were recorded by a blinded observer before genotyping.

Reporting for specific materials, systems and methods

We require information from authors about some types of materials, experimental systems and methods used in many studies. Here, indicate whether each material, system or method listed is relevant to your study. If you are not sure if a list item applies to your research, read the appropriate section before selecting a response.

Materials & experimental systems

n/a	Involved in the study	
☒	☐	Antibodies
☒	☐	Eukaryotic cell lines
☒	☐	Palaeontology and archaeology
☐	☒	Animals and other organisms
☒	☐	Clinical data
☒	☐	Dual use research of concern
☒	☐	Plants

Methods

n/a	Involved in the study	
☒	☐	ChIP-seq
☒	☐	Flow cytometry
☒	☐	MRI-based neuroimaging

Animals and other research organisms

Policy information about studies involving animals; ARRIVE guidelines recommended for reporting animal research, and Sex and Gender in Research

Laboratory animals	The zebrafish (D. rerio) wild-type strain TLEK (Tüpfel long fin/Ekkwill) is maintained in the animal facility of the Max Planck Institute of Immunobiology and Epigenetics, Freiburg, Germany and was used for crosses with the dnmt1, pole1, mcm10, and mat2aa mutants. The ikzf1:EGFP transgenic reporter line49 and the p53 mutant M214K were described previously; the p53(p.M214K) strain was obtained from M. Hammerschmidt.<br>FVB and B6 mice were used.
Wild animals	n/a
Reporting on sex	animals of both sexes were used
Field-collected samples	n/a
Ethics oversight	All zebrafish experiments were performed in accordance with relevant guidelines and regulations, approved by the review committee of the Max Planck Institute of Immunobiology and Epigenetics and the Regierungspräsidium Freiburg, Germany (license 35-9185.81/G-14/106; license 35-9185.81/G-15/115).<br>All mouse experiments were performed in accordance with the relevant guidelines and regulations, approved by the review committee of the Max Planck Institute of Immunobiology and Epigenetics and the Regierungspräsidium Freiburg, Germany (license AZ 35-9185.81/G-15/35; license AZ 35-9185.81/G-15/80; license AZ 35-9185.81/G-17/03).

Note that full information on the approval of the study protocol must also be provided in the manuscript.
